# Heterogeneity in the Returns to Credits for Public Two-Year College Entrants

**DOI:** 10.1007/s11162-021-09654-8

**Published:** 2021-08-06

**Authors:** Lauren Schudde, Meghan Shea

**Affiliations:** 1 Educational Leadership & Policy and Sociology, The University of Texas At Austin, George I. Sanchez Building 310A, 1912 Speedway D5400, Austin, TX 78712, USA; 2 Abt Associates, Cambridge, MA, USA

**Keywords:** Community college, Two-year college, College credits, Individual fixed effects, Labor market outcomes, Sub-baccalaureate, Interaction effects, Returns to higher education

## Abstract

Public two-year colleges offer an entry point to postsecondary education for many Americans who might otherwise forgo college. Most students leave college without a credential. A growing body of research examines the returns to higher education among two-year college entrants but primarily focuses on returns to credentials. This study examines the returns to different types of credits, including academic, technical, and developmental credits. In a series of individual fixed effects models, we use state administrative data following a population of public two-year college entrants to understand which college credits yield the greatest returns and how returns to credits vary across degree attainment. Our findings illustrate that average estimates of the returns to credits obscure varied patterns of returns among two-year college students, where sub-baccalaureate credential recipients appear to experience different returns to academic and technical credits compared with their peers.

Public two-year colleges offer an entry point to postsecondary education for many Americans who might otherwise forgo college. A growing literature examines the returns to higher education among two-year college entrants (e.g., Bahr et al., 2015; Hodara & Xu, 2016; Stevens et al., 2019; Xu & Trimble, 2016). Much of the extant research focuses on the returns to credentials. Yet the majority of college students, particularly those at two-year colleges, do not earn a credential (Snyder et al., 2018). In this study, we use state administrative data and individual fixed effects models to examine how different types of college credits, including academic, technical, and developmental credits, predict individual earnings and employment among public two-year college entrants and how those effects vary across degree attainment status. The analytic approach allows us to compare within the individual, examining how credit accrual predicts earnings while controlling for student characteristics.

Our study makes several contributions to the literature. First, we replicate extant research using individual fixed effects models to examine returns to different types of credits for community college entrants in a state with one of the highest postsecondary enrollments in the country—Texas. Second, we subsequently use a model with interaction terms to examine variation in credits returns by the highest degree earned, which allows us to uncover variation in the returns to credits across degree attainment (credential type and completion). Non-completers comprise the largest block of two-year colleges entrants; yet prior research focused on average returns for all students—controlling for degree attainment without examining variation in the relationship between credits and earnings across degree attainment.

## Returns to Education in the Public Two-Year Sector

A college education can yield substantial labor market benefits to students, contributing to individual status attainment. A credential can yield value above and beyond that of the accumulated years of education, where research suggests the returns to education are non-linear, with dramatic increases in years marking degree attainment—a phenomenon referred to as the “sheepskin effect” of education (Belman & Heywood, 1991). Sheepskin effects bolster support for theories that the *signal* of human capital, rather than accumulated skills, improves employment outcomes (Spence, 1973), though evidence regarding sheepskin effects in the returns to sub-baccalaureate credentials vary across gender and credential type (Bailey et al., 2004). College coursework should, theoretically, also improve labor market returns through accrued knowledge and skills and potentially by signaling those skills to prospective employers (Hodara & Xu, 2016; Monks, 2000; Rumberger & Thomas, 1993; Spence, 1973; Weisbrod & Karpoff, 1968; Weiss, 1995).

Before state administrative data were available, researchers worked to illuminate the returns to sub-baccalaureate degrees using national data. More recently, many scholars used population-level data from states to explore the returns to two-year colleges. Research in this field has traditionally focused on returns to credentials, but scholars recently argued for more research on the returns to credits given the high rate of non-completion among public two-year college entrants (Bahr, 2019; Zeidenberg et al., 2015). Next, we review the literature on the returns to credentials and credits among community college students.

## Returns to Credentials

Grubb (2002) reviewed the research on the returns of sub-baccalaureate degrees and coursework at community colleges, which, at the time, stemmed primarily from analyses using nationally representative data. Most estimates suggested a 20% to 30% boost in earnings for earning an associate degree compared with a high school diploma. Grubb also concluded that 1 year of coursework (without completing a degree) at either a two- or a four-year college increases an individual’s earnings by 5% to 10%. Bailey et al. (2004) leveraged three national data sets to examine the returns, in logged income, to postsecondary education. They found generally positive relationships between postsecondary educational attainment and employment outcomes but some variation in the relationship between sub-baccalaureate credential attainment and earnings. For example, they noted that attaining an associate degree positively predicts earnings for both men and women, but women who earned an occupational associate degree experienced a greater boost in earnings than women with 2 years of education (and no credential) in occupational programs. There appeared to be a sheepskin effect for occupational associate degrees for women, though this sheepskin effect was not present for an academic associate degrees or for certificates—this illustrates the usefulness of delineating between accumulated education and credential attainment and exploring variation across program type.

Access to state administrative data made it easier to link postsecondary transcript data to wage data, facilitating further inquiry into the returns to community college education. The nascent literature spans important, understudied topics, including the returns to certificates (Dadgar & Trimble, 2014; Xu & Trimble, 2016), career and technical education (Stevens et al., 2019), adult technical education (Carruthers & Sanford, 2018), developmental education (Hodara & Xu, 2016), community college education more generally and by major (Bahr, 2019; Bahr et al., 2015), and returns to college for marginalized sub-populations, like mothers receiving welfare (Turner, 2016). Much of the research relies on individual fixed effects approaches leveraging longitudinal data that tracks individuals with earnings prior to starting college.

The primary focus of this growing literature is on the returns to sub-baccalaureate credentials. Bahr et al. (2015) examined the returns to certificates and associate degrees in Michigan, finding that students received an earnings boost for short- and long-term certificates. Those gains were experienced primarily by men, whereas women saw higher returns to associate degrees than men; but everyone received a more substantial increase in earnings for having an associate degree compared with having no credential. Turner (2016) examined returns to community college among welfare recipients in Colorado. She found that career-oriented credentials led to larger returns than credentials that may facilitate transfer, like associate of arts degrees or related general studies programs.

Other researchers illustrated variation in returns to certificates. Dadgar and Trimble (2014) used data from Washington State to illustrate heterogeneous effects of certificates across program type. They identified negative effects of short-term certificates on earnings for women with null effects for men, but positive returns of long-term certificates for women and no significant returns for men. Xu and Trimble (2016) used a similar approach to illustrate positive returns to short- and long-term certificates in North Carolina and Virginia, where they observed varied returns across field of study. The highest long-term certificate returns in North Carolina went to students in Surgical Technology or Nursing, and the highest in Virginia went to students in Machine Shop or Automotive Repair and Analysis (with practical nursing coming in third) (Xu & Trimble, 2016). The differences across the two states suggest that context seems important for understanding the returns to sub-baccalaureate credentials.

Other scholars focused explicitly on vocationally focused sub-baccalaureate degrees. Using data from California, Stevens et al. (2019) examined the returns to Career and Technical Education (CTE) certificates and other sub-baccalaureate degrees. They illuminated average returns that ranged from 14 to 45%, where the highest returns were for students in healthcare fields. Jepsen et al. (2014) used data on community college students in Kentucky to illustrate sizeable returns to certificates, diplomas (long-term certificates, in this context), and AA degrees. They found positive returns to CTE associate degrees and diplomas for men, but minimal evidence for returns to the same degrees for women. Carruthers and Sanford (2018) focused on returns to adult technical education, leveraging data from Tennessee Colleges of Applied Technology (TCAT). They examined the returns to sub-associate credentials toward specific skills, primarily geared toward non-traditional, older students that attend part time. Students who received a TCAT diploma experienced a larger positive return to wages than did non-completers. The results illustrate the value of credential attainment, even in technical education, where skill development—potentially measured as courses taken/credits accrued—theoretically improves earnings.

## Returns to Credits

Many two-year college entrants leave college with credits but no credential in hand. Understanding the returns to credits, rather than just credentials, is particularly important in the two-year sector, where many students attend part-time, enroll in non-continuous patterns, and fail to finish a degree program (Belfield & Bailey, 2011; Zeidenberg et al., 2015). Using data from the Education Longitudinal Study of 2002, Marcotte (2019) found that, compared with individuals who received no college education, those who accrued any education at a community college experienced a 22% increase in earnings. He then explored variation in returns across credits, acknowledging heterogeneity among the community college students in terms of how much education they receive: some students accrue very few credits; others accrue a lot of credits but no credential; others earn their desired degree. He examined variation across 15-credit increments while controlling for highest degree and found a positive relationship between credits and earnings, although the observed patterns were not particularly strong.

In pursuing their educational aspirations, students at public two-year colleges take several different types of credits, including academic credits, technical credits, and developmental credits (required courses that must be taken prior to college-level coursework, in most settings, for students deemed not “college-ready”). For students planning to earn an academic (non-technical) associate or bachelor’s degree, accumulating academic credits is the most direct path to that goal. Students often start with general education courses—academic courses aimed at improving soft skills like critical thinking, communication, and quantitative reasoning. At most colleges, the general education core includes introductory courses distributed across broad fields like the humanities, social sciences, and physical sciences (Hart Research Associates, 2016; Jaschnik, 2016). General education courses comprise one third of the average college curriculum (Brint et al., 2009). Although academic credits may help students to pursue an academic degree (and by way of paving the path to that degree, promise high returns), it is unclear whether or how academic credits influence labor market outcomes. If the skills students accumulate through academic credits are easily presented on the job market, then academic credits could in fact lead to higher wages. However, if these skills are not easily perceived by potential employers, and the individuals must spend time out of the labor force to earn them, academic credits could in fact have negative implications for non-completers.

Many technical credits, which may also be referred to as vocational or CTE credits, typically apply toward requirements for technical/applied credentials (such as certificates or an associate of applied science) rather than for academic degrees (e.g. an associate or baccalaureate of arts). Technical coursework aims to provide contextual and hands-on learning to prepare students for real-world job opportunities (Holzer et al., 2013). The skills attained from these courses should, theoretically, apply seamlessly to related jobs. If the student and employer both value the attained skills (i.e., the program and its curriculum are appropriately aligned with demand in the labor market), it seems likely that technical credits offer positive returns on wages even without a credential. However, research suggests that many CTE programs are out of date and require revision to meet those expectations (Holzer et al., 2013). Although it is feasible that technical credits could lead to a big payoff, returns may vary by program and by context. Earning technical credits at a technical college may have some substantial labor market benefits over earning technical credits at a community college if technical colleges have stronger ties to potential employers (this may be true in Texas because technical colleges are fully funded using a performance-based funding model and have worked to align with local labor markets).

Developmental credits are offered to students whom colleges identify as academically underprepared for college-level coursework. Developmental course sequences delay students in taking college-level courses, increasing time and cost spent on a degree (Bettinger et al., 2013). If the coursework improves students’ skills sets, it is still possible that these credits could improve labor market outcomes, including earnings. Developmental English classes aim to improve English language proficiency and communication skills, which could improve labor market opportunities (McCabe, 2000); and some evidence suggests that dev-ed English credits boost individuals’ earnings (Hodara & Xu, 2016). Improving students’ basic numeracy skills could also theoretically improve employability and wages (McIntosh & Vignoles, 2001), but research suggests that dev-ed math may not produce those effects (Hodara & Xu, 2016), perhaps because of insufficiencies in how traditional developmental math pathways prepare students for math skills required in real-world jobs (Logue et al., 2016; Schudde & Keisler, 2019).

Compared with returns to credentials among public two-year college entrants, returns to credits have received less attention. Two extant studies offer the greatest insights. First, Bahr (2019) examined returns to cumulative credits across a large number of programs (including vocational programs) and degrees at California community colleges. He illustrated that the returns to credits vary between fields of study, with students in Public and Protective Services and Engineering Technology seeing the highest percentage change in their quarterly earnings. However, Bahr did not examine variation in the returns to different types of credits—developmental vs. academic vs. technical. Furthermore, although he simultaneously captured cumulative credits and credential earned (with non-completers as the reference), he did not explore whether the returns to credits vary across degree attainment, though he acknowledged the need to continue examining heterogeneity across a number of subgroups. He found the strongest returns to credits were from CTE fields, though it is difficult to know the role different types of credits might play in observed returns.

Second, Hodara and Xu (2016) focused on the returns to different types of credits, with a specific interest in how developmental credits influence wages for students in two large community college systems—North Carolina and Virginia. They estimated the returns to developmental math, developmental English, academic, and technical credits. Their results illuminate variation in the returns to developmental credit, where developmental English credits offered positive returns—potentially because of the increased likelihood of employment—but developmental math credits led to negative returns. Their models controlled for degree attainment but did not consider potential variation in credit returns across individuals who earn a credential and those who do not.

Despite the growing body of work on the returns to two-year college credits, the extant literature obscures how the returns to different types of college credits vary across degree attainment. Approximately 75% of public two-year college entrants never earn a degree (Snyder et al., 2018). Even non-completers are actively skill-building, where additional credits should increase human capital and therefore increase wages (Booth & Bahr, 2013). However, providing average estimates of the returns to credits for all college entrants may obscure varied patterns of credit returns, especially concerning given that there is some evidence of sheepskin effects among community college students (Bailey et al., 2004). Although the extant papers examining returns to public two-year credits control for degree attainment, examining heterogeneity in the returns to different types of credits across non-completers and completers (and various credential types) requires models with interaction effects. In our analyses, we explore heterogeneity in the returns to credits across degree status, anticipating that credit returns may differ across credential earned and completion status.

## Objective and Research Questions

To examine the returns to different types of public two-year college credits and how those returns vary based on degree status, we ask the following research questions:

1. How do different types of credits predict individual earnings and employment status, controlling for degree attainment?
2. How do the relationships between different types of credits and employment outcomes vary across degree attainment?
3. How do the patterns observed in research question 2 vary by major?

The first question replicates prior research focused on returns to education at two-year colleges, with an explicit focus on returns to different types of credits. The second research question allows flexibility in the returns to credits across degree status, anticipating that individuals may experience differential effects of credits based on whether they earn a credential and the type of credential earned. Finally, in the third research question, we consider whether those patterns vary across major.

## Methods

### Data

To examine the returns to college credits across community college entrants, we used state administrative data in Texas, the second largest college system in the country. The data were collected by the Texas Higher Education Coordinating Board (THECB) and the Texas Workforce Commission (TWC). We were provided data access through a restricted-use agreement with the Texas Education Research Center (ERC), a research center and data clearinghouse that holds longitudinal, student-level data in the state. The THECB data include information on student enrollment, demographics, credentials earned, and schedule data—transcript-level information about students’ course enrollment, grades, and credits—for all students attending public Texas postsecondary institutions. We merged the THECB student record data with the TWC’s Unemployment Insurance (UI) data, which provides quarterly earnings, using an assigned, anonymous ID number. Our derived data set includes quarterly earnings, college student enrollment records, credits, grades, and degree outcomes, along with demographic measures.

We focused on all first-time public two-year entrants in Texas in the 2011–2012 school year, the first entering cohort with THECB schedule data available to capture credit accumulation (N = 154,363). The data allowed us to follow each student through the most recent data release for the 2017–2018 school year (7 years of follow-up). We used UI data starting from 3 years prior to enrollment—quarter 4 of 2008—through the most recent UI data—quarter 2 of 2018—to capture individuals’ earnings over 9 years.

### Sample Restrictions

Following past literature (Bahr et al., 2015; Belfield & Bailey, 2017; Hodara & Xu, 2016; Xu et al., 2016), we restricted our sample to students who entered college at 18–65 years of age (N = 138,414). Individuals who begin college before they are 18 years old probably do not have any earnings data prior to enrollment, or if they do, it would probably be from summer or part-time work that is not reflective of potential future earnings. Individuals who enroll for the first time after the age of 65 have probably already peaked in their earnings profile and are not enrolling to improve future job prospects or earnings. We also restricted the sample to students with at least some earnings in the three years prior to enrollment (N = 95,330) and with any positive earnings in our sample period (N = 92,850) in order to compare earnings—within an individual’s own earning trajectory—over time. Restricting to students with prior earnings substantially reduced the analytic sample, but was necessary to use our analytic strategy; ultimately, it means the analytic sample is only generalizable to community college entrants with prior experience working for pay. We also focused on students with complete schedule data, so we could capture their credit accrual; these restrictions resulted in our final analytic sample of 91,759 public two-year college entrants who were aged 18–65, had prior earnings and complete schedule data, and were first-time college entrants. The analytic sample included students who attended 72 community college campuses (within 50 community colleges/districts^1^) and 4 technical college campuses (within one technical college system comprised of campuses that report separately to the THECB).

### Variable Selection

THECB schedule data capture students’ course enrollments and credits earned by term. The course numbering system used by community and technical colleges in the state allowed us to delineate several types of earned credits. We identified five types of credits—academic, technical credits from a technical college, technical credits earned at a community college, developmental math, and developmental English—using the course number and, in some cases, the type of two-year institution where the credits were taken. We identified academic credits (college-level academic-focused credits) using course numbers in the state’s Academic Course Guide Manual (ACGM), which specifies which courses count toward academic degrees at public universities and at community and technical colleges. Community and technical colleges in Texas are required to offer only courses listed in either the ACGM or the Workforce Education Course Manual (WECM), which specifies which courses are approved in various technical fields (THECB, 2014). We identified technical credits using the WECM and distinguished between two types of technical credits—those taken at technical colleges and those taken at non-technical colleges—because we anticipated differences in returns of technical credits earned at different types of institutions (the technical colleges in the state are funded based on labor market returns, so we expected they may have different returns than technical credits from community colleges). Finally, we identified developmental education credits using course numbers and prefixes that align with those listed as developmental education in the ACGM and distinguished between math and English dev-ed coursework, following the example of prior literature (Hodara & Xu, 2016).

The enrollment data provided us with information about when (semester, year) and where (FICE codes) students enrolled, enabling us to control for terms in which individuals enrolled at community and technical colleges or public universities within the state. It also allowed us to capture students’ last known college major, using the first two-digits of CIP codes, which we used to examine variation in credit returns across majors in research question 3. Using data from the graduation file, we captured the types of credentials earned, including certificates, associate degrees, and bachelor’s degrees, and the number of terms since credential attainment.

We used two outcome variables: (1) quarterly earnings in terms where a student has positive earnings (i.e., we exclude terms without earnings and include terms with earnings^2^), derived from the TWC data; (2) employment status, a dichotomous measure derived from the TWC data, in which students are deemed employed if they have any earnings and not employed when missing quarterly earnings in a given term. The measure of earnings is capped at the first and 99th percentiles to address outliers in the reported quarterly earnings.^3^ Given our reliance on quarterly earnings from the UI data, our earnings outcome is conditional on employment as captured by the TWC UI data. UI data is only collected for employment in which employees contribute unemployment insurance, which means someone working for pay in a position that does not pay into unemployment will appear as having no earnings in that term; we also only have UI data for the state of Texas (for further discussion, please see “Limitations” section). Because the UI data provided to the ERC primarily includes quarterly wages, we were unable to include other employment outcomes, like hours worked or an indicator of working part vs. full time. To perform individual fixed effects, we used quarterly earnings and employment data for all individuals before their initial enrollment in college, during enrollment, and after enrollment. The enrollment and schedule data from the THECB were captured by semester, so we converted quarterly workforce outcome variables to match school semesters as closely as possible. We matched quarter 1 with semester 2 (spring) of the school year, quarter 2 with semester 3 (summer), and the average earnings of quarters 3 and 4 with semester 1 (fall), following examples from prior research (e.g., Hodara & Xu, 2016; Xu & Trimble, 2016).

Table 1 presents a description of the variables included in our model. Because the model—described next—controlled for individual fixed effects, we did not need to include individual background measures, like race or indicators of prior achievement, and instead focused on variables that change over time and are likely to predict employment and earnings outcomes. Most importantly, we included various types of credits accumulated over time in order to examine how they predict employment and earnings.

### Analytic Strategy

We employed individual fixed effect models to examine the effects of credit accumulation on employment outcomes (earnings and employment status). Although using a fixed effects model involves stricter data requirements, the model has benefits over a traditional regression approach that is susceptible to omitted variable bias—such as allowing researchers to control for within-person characteristics that are typically unobserved and/or difficult to measure (Belfield & Bailey, 2017). To use the approach, we leveraged panel data that follows individuals for 3 years (12 quarters, which translates to 9 academic terms) before their first semester of enrollment and 7 years (28 quarters or 21 academic terms) after their first enrollment. Because we expected that different types of credits would affect earnings outcomes differently, we broke out accumulated credits into academic credits, technical credits earned at a technical college, technical credits earned at a non-technical college, developmental math credits, and developmental English credits.

### Model 1: Replicating Prior Research

We begin with a model on returns to different types of credits similar to extant research. Equation (1) presents Model 1:

(1) $${EmploymentOutcome}_{it}=\alpha_{i}+\theta_{i}t+\beta_{1}{AcademicCredits}_{it}\;\;+\beta_{2}{TechatTechCredits}_{it}+\beta_{3}{TechatCommCredits}_{it}\;\;+\beta_{4}{DevMathCredits}_{it}+\beta_{5}{DevEngCredits}_{it}+{Enroll}_{it}\;\;+{EnrolledCredits}_{it}+{Awards}_{it}+{Awards}_{it}\;*\;{TimeSinceAward}_{it}+\lambda_{t}+A_{it}+\gamma_{it}+\mu_{it}$$

where ${\text{EmploymentOutcome}}_{it}$ represents either individual i’s quarterly earnings or their employment status in time t.^4^ We have a term for total academic credits earned prior to time $t,\;{AcademicCredits}_{it}$, in addition to terms for all other credit types earned prior to time t. We can then interpret $\beta_{1}$ as returns to non-developmental academic credits, $\beta_{2}$ as returns to technical credits earned at a technical college, $\beta_{3}$ as returns to technical credits earned at a community college, $\beta_{4}$ as returns to developmental math credits, and $\beta_{5}$ as returns to developmental English credits. The model includes individual fixed effects, $\alpha_{i}$, to capture within-person changes over time before and after enrollment. We also include individual specific trends, $\theta_{i}t$, to capture unobservable characteristics that are correlated with success and change at a constant rate over time. Dynarski et al. (2018) showed the importance of including these individual time trends in individual-level fixed effect models estimating returns to two-year college degrees.

When students are actively enrolled in college, their earnings and employment status may not reflect their returns to schooling if they do not work full time and/or if they work for little pay. To control for this temporary dip along the way from pre-college earnings to post-college earnings, we included current enrollment for individual i at time t through indicator ${Enroll}_{it}$. We also controlled for the intensity of enrollment with ${EnrolledCredits}_{it}$, which captures how many credits individual i enrolled in during quarter t. Conceivably, if an individual is enrolled in only 3 credits, their earnings might not be as affected as if they are enrolled in 18 credits. $Awards_{it}$ is a vector of dichotomous indicators for any award earned (one for a certificate, one for an associate degree, and one for a bachelor’s degree) prior to time t, where “no degree” (non-completion) serves as the reference category. By including this vector, we controlled for the impact of each degree received on wages. We also include a term,$Awards_{it}*\;{TimeSinceAward}_{it}$, that interacts the awards vector with an indicator for the time since earning the award, to account for the accumulating effect of degree receipt on earnings.^5^ The term $\lambda_{t}$ captures the type of term, absorbing variation that may occur across fall, spring, and summer terms (summer is the reference). To address a potential drop in earnings prior to college enrollment (referred to in the literature as an Ashenfelter’s Dip), we included indicators for each term in the year prior to college entrance, represented by $A_{it}$. We also capture potential geographic changes in labor market opportunities by including a county fixed effect, $\gamma_{it}$—because our model compares individuals to themselves over time, the county-level fixed effect ultimately captures variation among individuals that change their county of employment over time. Finally, $\mu_{it}$ represents the error term.

### Model 2: Heterogeneity Across Degree Status

Our initial model accounts for degrees earned, but, based on the literature, we anticipate that returns to credits may vary based on credential attainment. In our second model, we explore heterogeneity in returns to different types of credits across degree attainment and enrollment status. Equation (2) presents Model 2:

(2) $${EmploymentOutcome}_{it}=\alpha_{i}+\theta_{i}t+\beta_{1}{AcademicCredits}_{it}\;\;+\beta_{2}{TechatTechCredits}_{it}+\beta_{3}{TechatCommCredits}_{it}\;\;+\beta_{4}{DevMathCredits}_{it}+\beta_{5}{DevEngCredits}_{it}\;\;+\delta_{1}{AcademicCredits}_{it}\;*\;{Enrolled}_{it}\;\;+\delta_{2}{TechatTechCredits}_{it}\;*\;{Enrolled}_{it}\;\;+\delta_{3}{TechatCommCredits}_{it}\;*\;{Enrolled}_{it}\;\;+\delta_{4}{DevMathCredits}_{it}\;*\;{Enrolled}_{it}\;\;+\delta_{5}{DevEngCredits}_{it}\;*\;{Enrolled}_{it}\;\;+\gamma_{1}{AcademicCredits}_{it}\;*\;{HighestAward}_{it}\;\;+\gamma_{2}{TechatTechCredits}_{it}\;*\;{HighestAward}_{it}\;\;+\gamma_{3}{TechatCommCredits}_{it}\;*\;{HighestAward}_{it}\;\;+\gamma_{4}{DevMathCredits}_{it}\;*\;{HighestAward}_{it}\;\;+\gamma_{5}{DevEngCredits}_{it}\;*\;{HighestAward}_{it}\;\;+\xi_{1}{AcademicCredits}_{it}\;*\;{HighestAward}_{it}\;*\;{Enrolled}_{it}\;\;+\xi_{2}{TechatTechCredits}_{it}\;*\;{HighestAward}_{it}\;*\;{Enrolled}_{it}\;\;+\xi_{3}{TechatCommCredits}_{it}\;*\;{HighestAward}_{it}\;*\;{Enrolled}_{it}\;\;+\xi_{4}{DevMathCredits}_{it}\;*\;{HighestAward}_{it}\;*\;{Enrolled}_{it}\;\;+\xi_{5}{DevEngCredits}_{it}\;*\;{HighestAward}_{it}\;\;\;*\;{Enrolled}_{it}+{Enrolled}_{it}\;*\;{HighestAward}_{it}+{Enrolled}_{it}\;\;+{HighestAward}_{it}+{HighestAward}_{it}\;*\;{TimeSinceAward}_{it}+\lambda_{t}+A_{it}+\gamma_{it}+\mu_{it}$$

where ${HighestAward}_{\text{it}}$ is a categorical indicator of the highest degree earned prior to time t (with a reference category of non-completion) and ${Enrolled}_{it}$ is a dummy for whether a student is enrolled in time t, which allows us to account for the temporary lost wages while the student is enrolled. Whereas Model 1 included a vector of dichotomous indicators for each award earned prior to time t, Model 2 simplifies this vector into one categorical indicator for the highest degree earned prior to time $t,\;{HighestAward}_{it}$. Unlike Model 1, Model 2 allows for returns to different types of credits to vary across degree status (highest award)—this is captured through an interaction between each credit measure and ${HighestAward}_{it}$. We also allow that to vary based on enrollment status (a three-way interaction). For example, in Model 2, the effects of academic credits may differ based on whether an individual earned an associate degree, beyond just the influence of the degree itself, and by current enrollment status.

Our approach in Model 1 is similar to that used in prior research (Hodara & Xu, 2016), where we can obtain a weighted average of the credit coefficients for each credit type in the model across individuals who receive different types of credentials [Bahr (2019) used a similar approach, but did not distinguish between credit types]. The approach estimates the direct relationship between credits and employment outcomes but assumes it is the same for people who have a credential and for those who do not. In Model 2, we relax that assumption, allowing for variation in the returns to different types of credits across different degree statuses—including credential type and non-completion.

### Limitations

Individual fixed effects models leverage panel data to estimate the individual-level increase in earnings between time periods (in our case, academic terms); by comparing earning trajectory within the individual, the models difference out individual characteristics that are unobservable and time invariant (Belfield & Bailey, 2017, Scott-Clayton & Wen, 2019). Belfield and Bailey (2017) describe two major drawbacks of the approach: (1) the models require strong assumptions about time trends and earnings trajectories of students during and after college and results may be sensitive to model specifications; (2) the models require limiting the analytic sample of students due to the need for data before, during, and after college (e.g., restricting to those with prior work experience), which can bias results.

Related to the first point, results from individual fixed effects models may be susceptible to bias if the trajectory of returns varies across degree attainment type (e.g., the earnings trajectory of associate degree earners looks different than the earnings trajectory of non-completers) (Belfield & Bailey, 2017). Research suggests that it *is* likely that earning trajectories differ across degree attainment (and, in our case, we would also anticipate they differ across type of credits accrued) (Belfield & Bailey, 2017; Dynarski et al., 2018; Minaya & Scott-Clayton, 2017). Dynarski et al., (2018) demonstrated how accounting for pre-college dips in earnings, earnings trends during college, and differential earnings growth after college can reduce the bias induced by that heterogeneity. Following their recommendation, based on work by Jacobson et al. (2005), we include individual time trends in our models to control for unobserved individual factors that change at a constant rate over time (e.g., age, work experience). Capturing “individual-specific heterogeneity” in earnings trajectories should further address selection bias in estimating returns to credits (Hodara & Xu, 2016). However, as described in Hodara and Xu (2016), if students who primarily earn one type of credit follow different wage trajectories than peers who primarily earn another type of credit, both Model 1 and Model 2 may not effectively address that issue. Model 2, which focuses on heterogeneity across type of credits and degree attained, attempts to parse out some of that heterogeneity. We anticipate (and model) differential returns to types of credits across degree attainment status by including interaction terms to capture that variation. However, the individual fixed effects approach is best suited for comparing time periods when individuals have an award to time periods in which they do not. We do not anticipate that the individual fixed effects approach can entirely address selection into different credit types. For that reason, our results should be thought of as correlations, not causal effects.

Related to the second point about sample restrictions based on missing data, estimating our employment outcomes based on UI records has important implications. In the data, people who work out of the state or in a position that does not pay into unemployment insurance are indistinguishable from individuals who remain in the state but are not working; all of those cases would show up as having missing earnings—captured as being unemployed—in a given term. Recent research comparing survey and state administrative data found that despite the obvious limitations of state UI data, missing UI data in state data sets does not appear to influence the results in a meaningful way, where the broad patterns of estimated returns in state administrative data compared with nationally representative data are consistent (Scott-Clayton & Wen, 2019). Similarly, Xu and Trimble (2016) found that restricting UI records to within state (compared to capturing UI records from neighboring states) results in a similar pattern of effects for the returns to certificates, where—if anything—the restriction risks slightly underestimating returns. Ultimately, in using Texas ERC data, it is necessary to restrict the sample to individuals with quarterly earnings records in the UI data. As noted above, our results should be interpreted as generalizable to those with earnings (as captured in the UI data) prior to college enrollment and at least some earnings during the subsequent terms.

## Results

### Descriptive Statistics

Table 2 presents descriptive statistics for our full analytic sample of public two-year entrants (first column) and by highest degree earned: bachelor’s degree recipients, associate degree recipients, certificate recipients, and non-completers. About 33% of Texas public two-year college entrants in 2011 were White, 19% Black, and 42% Hispanic. Slightly more than half of public two-year college enrollees identified as women. About half of the students in the full analytic sample took at least one developmental education course in math (51%), and slightly fewer took developmental English (48%). Although a large portion of the full sample of students (38%) took some technical credits, very few (2%) ever attended technical colleges—meaning community colleges were providing the bulk of the technical coursework.

In the full analytic sample, individuals accrued, on average, 44.71 total credits (*SD* = 42.62). The high variation in average credits earned appeared to be driven by dramatic variation (as we would expect) in credits accrued across degree type and completion status. An associate degree requires at least 60 credits, though the certificate programs in Texas require far fewer (ranging from 19 to 36 credits for a level 1 certificate and slightly higher for a level 2 certificate). On average, bachelor’s degree recipients earned 132.30 credits (*SD* = 29.21) and associate degree recipients earned 103.26 credits (*SD* = 30.44). Among non-completers (column 5), students earned an average of 31.26 credits total (*SD* = 30.46), where those credits largely comprised academic credits (mean = 21.34, *SD* = 28.13) and fewer technical credits (mean credits at technical colleges = 0.26, *SD* = 3.01; mean credits at community colleges = 3.64, *SD* = 8.80). The number of developmental math and English credits was almost even across the analytic samples, though degree recipients took slightly fewer developmental English credits than non-completers. Overall, approximately 6% of the entire sample earned a certificate, 12% an associate degree, and 5% a bachelor’s degree.

Among public two-year college entrants, 81% did not earn any award within 7 years of entrance. Their descriptive statistics are presented in the final column of Table 2. Compared with students who earned a bachelor’s degree, non-completers were less likely to be White, more likely to be Black or Hispanic, and about 2.5 years older at college entrance. Compared with students who earned a credential, non-completers appeared more likely to take developmental English and, other than associate degree earners, were among the most likely to take developmental math. Students who earned a certificate as their highest credential accrued the most technical credits. Non-completers accumulated fewer credits per semester enrolled (mean = 7.78, *SD* = 3.67) compared with bachelor’s awardees (mean = 10.97, *SD* = 4.02), associate degree earners (mean = 9.19, *SD* = 3.80), or certificate earners (mean = 9.57, *SD* = 4.52). The observed variation in student background and credit accrual bolsters support for our use of individual fixed effects while controlling for individual trends, which allows us to compare individuals with themselves over time.

### Returns to Different Types of Credits, Controlling for Degree Attained

Table 3 shows the results for Model 1, presenting the average returns to different types of credits among public two-year college students while controlling for degrees earned. In interpreting results from the first column (where the dependent variable is the natural log of quarterly wages), we exponentiate the coefficients to more precisely estimate percentage change in quarterly wages, in line with previous literature (Marcotte, 2019; Stevens et al., 2019). We find that different types of credits accrued at a public two-year college appear to play different roles in the earnings. For each additional academic credit earned, students experience a decrease in average quarterly earnings of about 0.3% (β = − 0.003, *SE* = 0.000, *p* < 0.001)—this translates roughly to a 0.9% decrease in wages for each additional 3-credit academic course taken. Developmental credits, on the other hand, are associated with positive returns, on average. Each additional developmental English credit predicts an increase in earnings of 1.3%, and each additional developmental math credit predicts an improvement of 1.4% (dev-ed English: β = 0.013, *SE* = 0.001, *p* < 0.001; dev-ed math: β = 0.014, *SE* = 0.001, *p* < 0.001). We also find small positive returns to technical credits—where each additional technical credit at a technical college is associated with an increase of 0.7% in average quarterly wages and each additional technical credit at a community college is associated with an increase of 0.4% (tech credits from technical colleges: β = 0.007, *SE* = 0.001, *p* < 0.001; tech credits from community colleges: β = 0.004, *SE* = 0.000, *p* < 0.001).

The second column in Table 3 shows results for employment status, where coefficients represent the association between the independent variable and the probability of employment. For each additional academic credit earned, students experience a 0.3-percentage-point decrease in the probability of employment (β = − 0.003, *SE* = 0.000, *p* < 0.001). Each additional developmental English and developmental math credit predicts a 1.2 and 1.3-percentage-point increase in the probability of employment, respectively (dev-ed English: β = 0.012, *SE* = 0.000, *p* < 0.001; dev-ed math: β = 0.013, *SE* = 0.000, *p* < 0.001). We also find a small positive relationship between technical credits and employment, where each additional technical credit is associated with between a 0.2- and 0.3-percentage-point increase in the probability of employment (tech credits from technical colleges: β = 0.003, *SE* = 0.000, *p* < 0.001; tech credits from community colleges: β = 0.002, *SE* = 0.000, *p* < 0.001).

The results from Model 1 also suggest that earning a degree offers large returns, above and beyond the returns generated by credits earned. Compared with no credential, a bachelor’s degree is associated with a 46.2% increase in quarterly wages and 12.5-percentage-point increase in the probability of employment (log wages: β = 0.380, *SE* = 0.015, *p* < 0.001; employment: β = 0.125, *SE* = 0.008, *p* < 0.001). An associate degree and a certificate predict a boost quarterly wages by 2.2% and 13.7% respectively, where earning a certificate is also linked to an increased probability of employment (log wages: associate degree: β = 0.022, *SE* = 0.009, *p* < 0.05; certificate: β = 0.128, *SE* = 0.011, *p* < 0.001). All credential recipients had higher returns to wages than non-completers, though associate degree earners received the smallest boost from their credential and did not experience an increase in their probability of employment. The model does not account for variation in the returns to credits based on degree earned.

### Returns to Different Types of Credits, Allowing Variation Across Degree Status

**Fig. 1:**
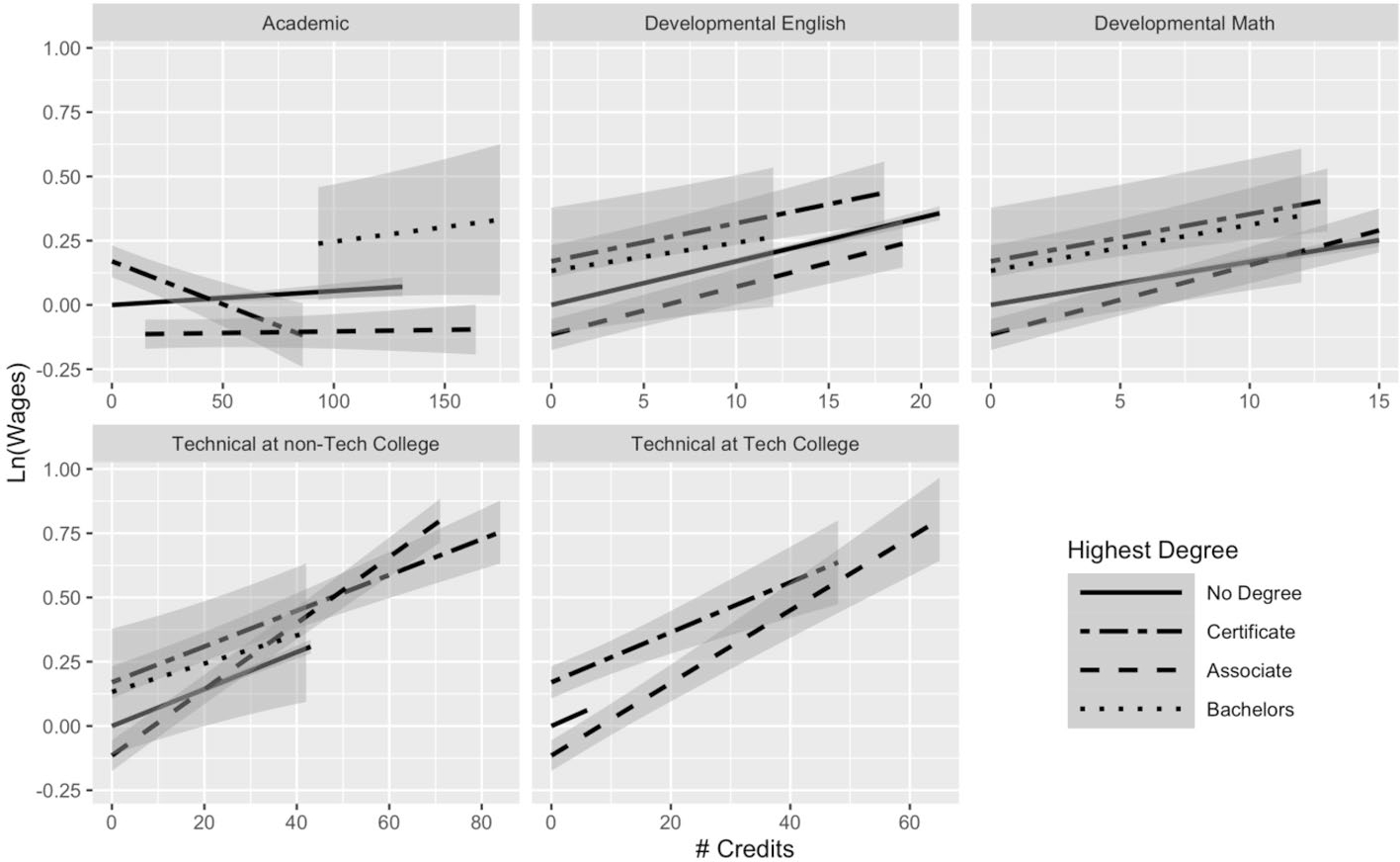
Average returns to different types of credits by highest degree for students no longer enrolled: Model 2 results for earnings. N(students) = 91,759; N(student-semesters) = 1,799,636. This figure presents predicted earnings (in logged quarterly earnings) with 95% confidence intervals, obtained using the results from Model 2 for students not currently enrolled. For full regression results, see Online Appendix A, Table A1 (this figure makes use of the interaction terms)

Using Model 2, we build a fuller picture of how credentials moderate returns to credits. Figure 1 plots the returns on wages to academic, developmental, and technical credits across highest degree earned for students who are no longer enrolled in college. In Model 2, these relationships were captured using three-way interactions terms (full regression results are available in Online Appendix A, Table A1). We find that both type of credit earned and degree attainment (including non-completion and type of degree) are important for understanding the returns to credits on earnings. In the first panel in Fig. 1 (top left corner), we observe that non-completers, associate degree recipients, and bachelor’s degree recipients experience small positive returns to each additional academic credit (captured by a positive slope). Non-completers received a 0.05% increase in wages per academic credit, compared with a 0.01% increase for associate degree recipients and a 0.11% increase for baccalaureate recipients (see Table A1 in Online Appendix^6^). The returns to each additional credit for certificate earners are negative, as illustrated by the slope of the line, where they experience, on average, a 0.33% decrease in wages per academic credit.^7^

**Fig. 2:**
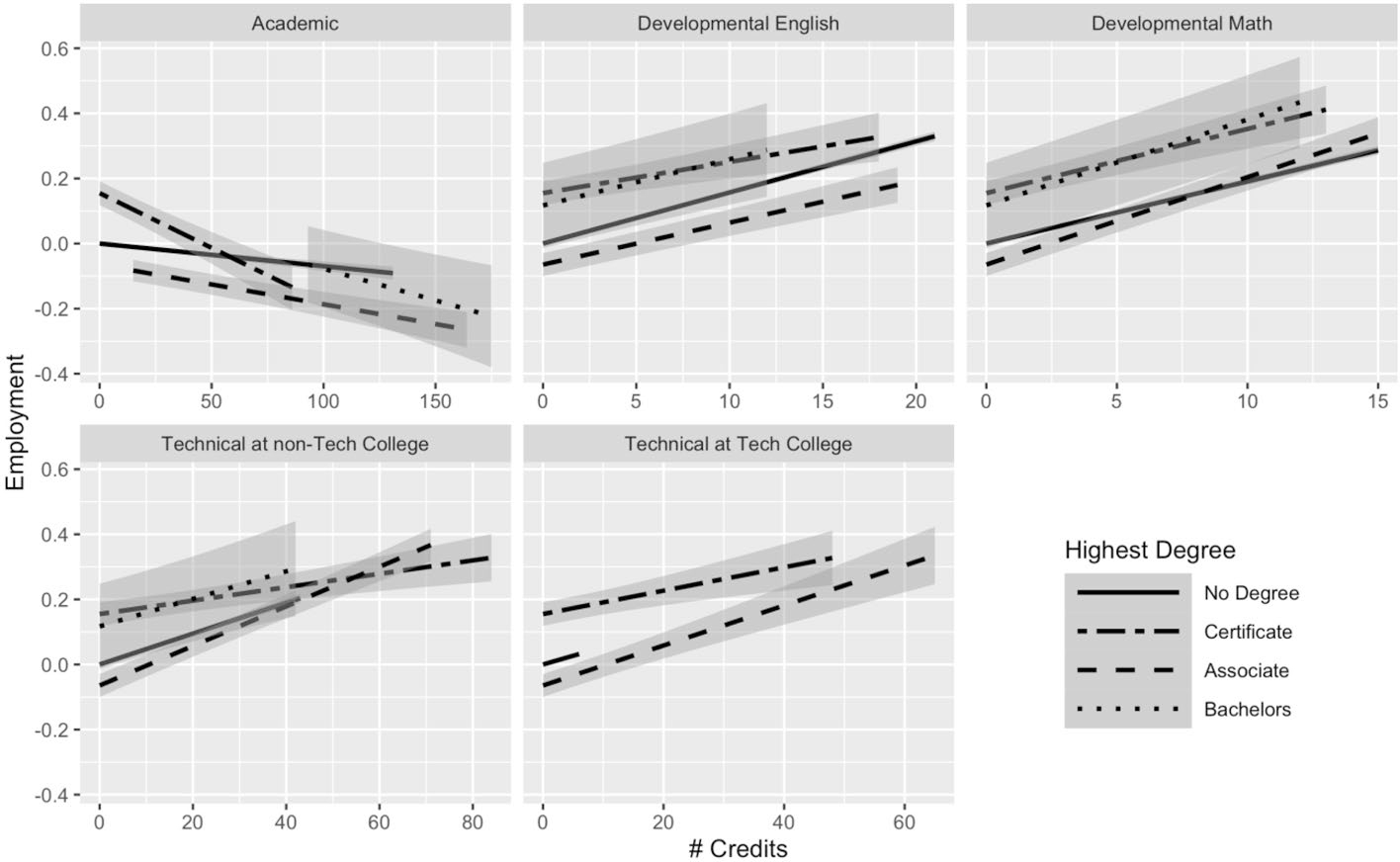
Average returns to different types of credits by highest degree for students no longer enrolled: Model 2 results for employment status. N(students) = 91,759; N(student-semesters) = 2,661,011. This figure presents predicted employment with 95% confidence intervals, obtained using the results from Model 2 for students not currently enrolled. For full regression results, see Online Appendix A, Table A1 (this figure makes use of the interaction terms)

Figure 2 plots the returns on employment to academic, developmental, and technical credits across highest degree earned for students who are no longer enrolled in college. Unlike the returns to wages, where most of the returns are positive, the relationship between academic credits and employment is negative across all degree attainment statuses. For each additional academic credit, non-completers experience a 0.07-percentage-point decrease in the probability of employment; similarly, certificate earners experience a 0.33-percentage-point decrease, and associate and bachelor’s degree recipients see a 0.12- and 0.19-percentage-point decrease in the probability of employment.^8^

For all other types of credits—technical credits at a technical college, technical credits at a community college, developmental math credits, and developmental English credits—the return to each additional credit is positive for both wages and employment, but the slopes differ dramatically for some types of credits. For instance, the positive per-credit returns on both wages and employment for developmental English credits appear similar across degree attainment status—the slopes are quite similar. For technical credits, this is not the case, particularly for technical credits earned at community colleges (more students earned technical credits from non-technical colleges than from technical colleges, as shown in Table 2, probably because the technical college system in Texas is quite small). The lower left panel of Fig. 1 shows the returns for wages to technical credits at non-technical colleges, where associate degree recipients appear to receive the highest per-credit boost to technical credits (though they have a lower starting point) compared with all the other groups, which have flatter (though positive) slopes. Figure 2 shows that associate degree recipients also appear to receive the highest per-credit boost on employment to technical credits, with non-completers in a close second, while certificate recipients have the flattest slope (lowest returns to employment for each additional credit).

For individuals who earn an associate degree or a certificate, it appears that additional technical credits, both at technical colleges and at community colleges, provide the largest per-credit increase in wages. For example, associate degree recipients experienced an increase in wages of 1.4% and 1.3% for each additional technical credit at a technical college and at a community college, respectively. This also holds for probability of employment; associate degree recipients see a 0.6-percentage-point increase in the probability of employment, on average, for each additional technical credit at a technical college or community college. Although the slopes give us a sense of the per-credit boost received by individuals in different degree attainment statuses, we also see that degree attainment, particularly baccalaureate attainment, influences the baseline for individual earnings and for probability of employment. Bachelor’s degree earners tend to have higher quarterly earnings and probability of employment than the other groups across the credits attained (this is particularly apparent in the academic credit plot in Fig. 1).

All of the patterns presented for Model 2 up to this point apply to individuals no longer enrolled in college. We expect that individuals no longer enrolled are more likely to be in the labor force full time and experiencing returns to their schooling, compared with students who are still enrolled in college. The results for currently enrolled students look quite different from the results for those no longer enrolled (see Figures B1 and B2 in Online Appendix B for the results for students still enrolled in college). The returns are much smaller across the board, which is not surprising since more of these individuals may be working part time.

### Variation by Major

**Fig. 3:**
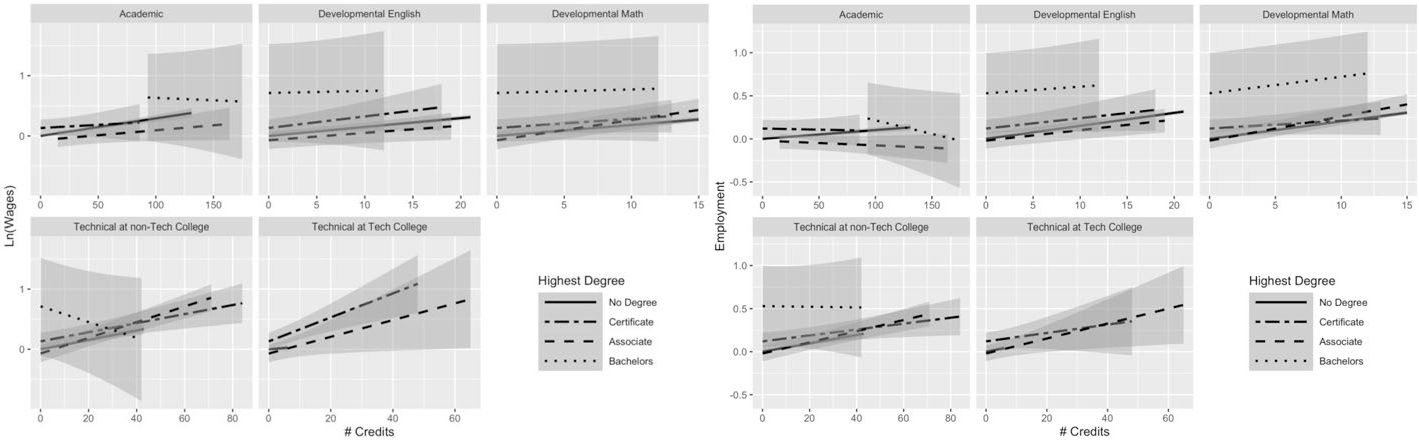
Liberal arts majors: average returns to different types of credits by highest degree. This figure presents predicted earnings (in logged quarterly earnings) (left panel) and predicted employment (right panel) with 95% confidence intervals, obtained using the results from Model 2 for students not currently enrolled. The analysis was run on a subgroup of students who were identified as having a liberal arts major. For full regression results, see Online Appendix C, Table C1 (this figure makes use of the interaction terms). For regression on earnings; N(students) = 31,859; N(student-semesters) = 625,910. For regression on employment: N(students) = 31,859; N(student-semesters) = 923,911

**Fig. 4:**
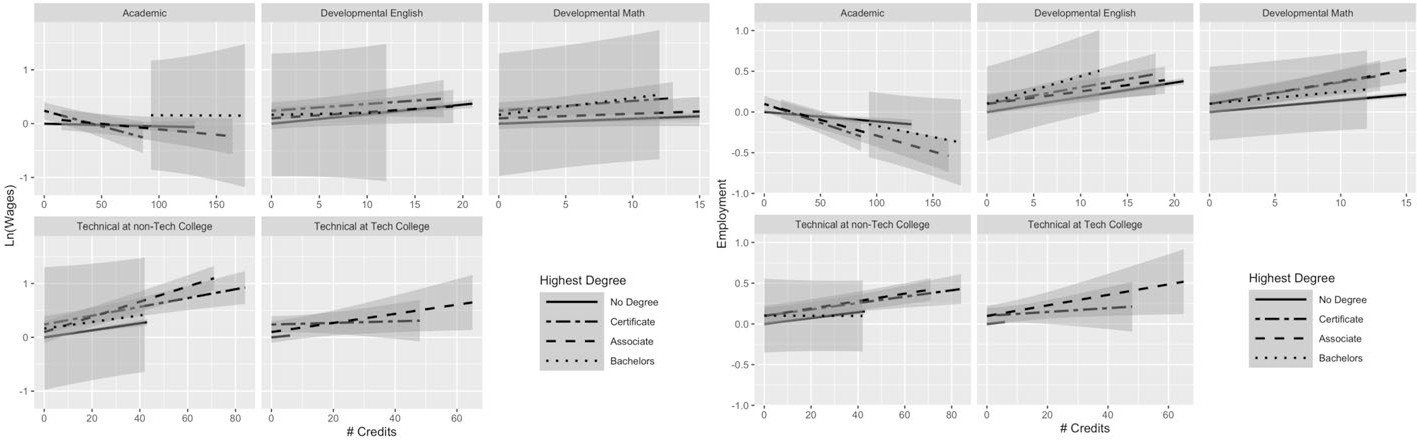
Health professional majors: average returns to different types of credits by highest degree. This figure presents predicted earnings (in logged quarterly earnings) (left panel) and predicted employment (right panel) with 95% confidence intervals, obtained using the results from Model 2 for students not currently enrolled. The analysis was run on a subgroup of students who were identified as having a health professions major. For full regression results, see Online Appendix C, Table C2 (this figure makes use of the interaction terms). For regression on earnings: N(students) = 12,148; N(student-semesters) = 244,409. For regression on employment: N(students) = 12,148; N(student-semesters) = 352,292

**Fig. 5:**
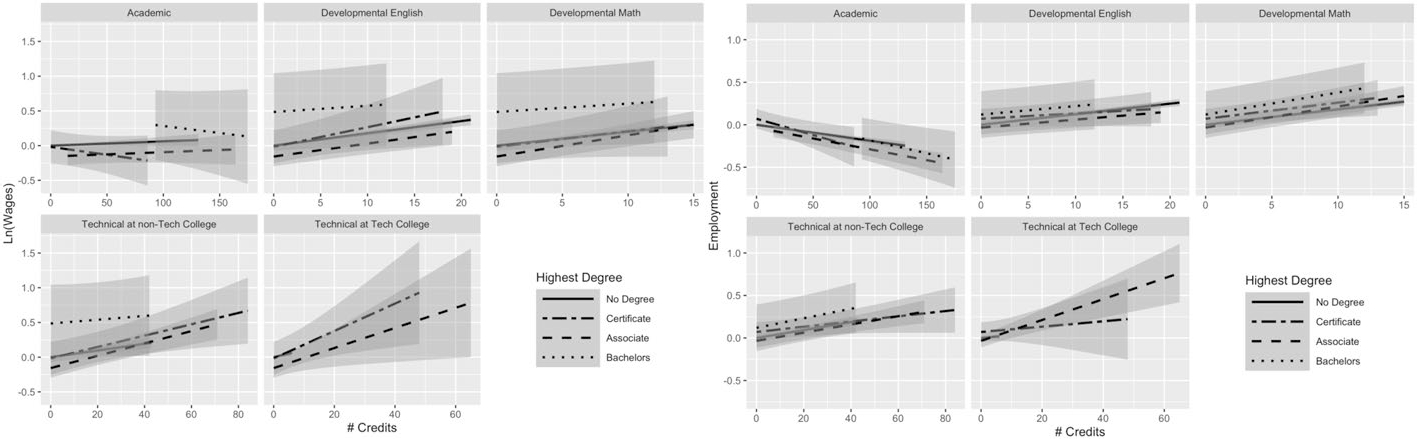
Business majors: average returns to different types of credits by highest degree. This figure presents predicted earnings (in logged quarterly earnings) (left panel) and predicted employment (right panel) with 95% confidence intervals, obtained using the results from Model 2 for students not currently enrolled. The analysis was run on a subgroup of students we identified as having a business major. For full regression results, see Online Appendix C, Table C3 (this figure makes use of the interaction terms). For regression on earnings: N(students) = 11,056; N(student-semesters) = 222,383. For regression on employment: N(students) = 11,056; N(student-semesters) = 320,624

**Fig. 6:**
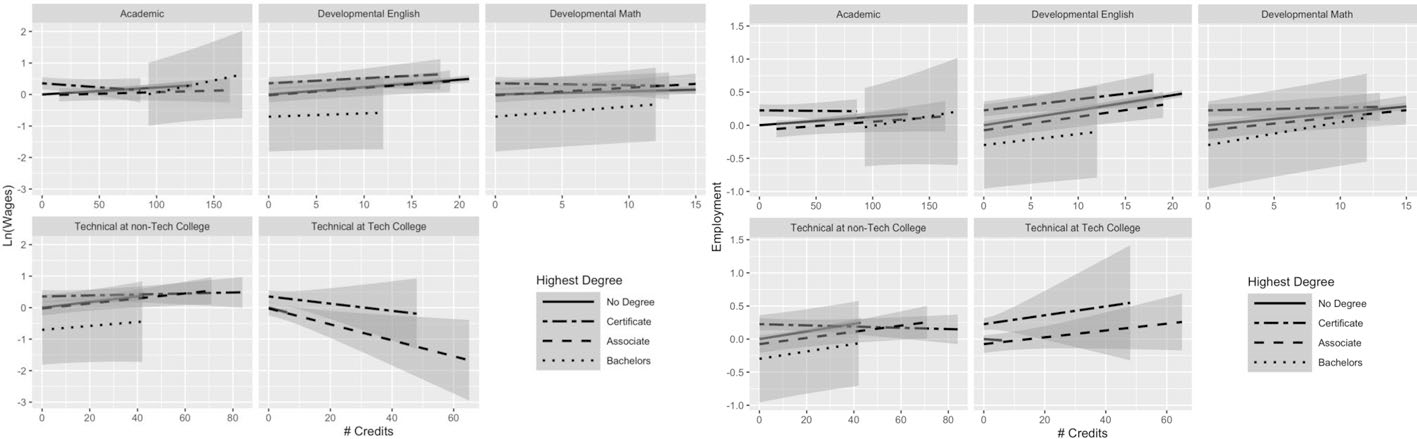
Law enforcement majors: average returns to different types of credits by highest degree. This figure presents predicted earnings (in logged quarterly earnings) (left panel) and predicted employment (right panel) with 95% confidence intervals, obtained using the results from Model 2 for students not currently enrolled. The analysis was run on a subgroup of students we identified as having a law enforcement major. For full regression results, see Online Appendix C, Table C4 (this figure makes use of the interaction terms). For regression on earnings: N(students) = 5,087; N(student-semesters) = 102,022. For regression on employment: N(students) = 5,087; N(student-semesters) = 147,523

**Fig. 7:**
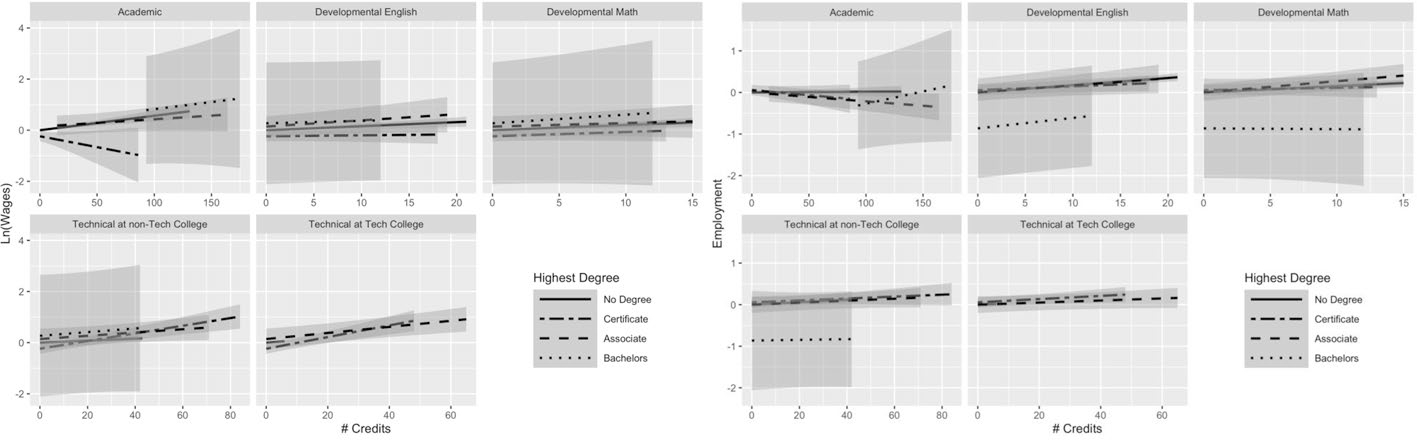
Engineering technology majors: average returns to different types of credits by highest degree. The figure presents predicted earnings (in logged quarterly earnings) (left panel) and predicted employment (right panel) with 95% confidence intervals, obtained using the results from Model 2 for students not currently enrolled. The analysis was run on a subgroup of students we identified as having an engineering technology major. For full regression results, see Online Appendix C, Table C5 (this figure makes use of the interaction terms). For regression on earnings: N(students) = 2,705; N(student-semesters) = 54,376 For regression on employment: N(students) = 2,705; N(student-semesters) = 78,445

Figures 3, 4, 5, 6 and 7 help us understand whether the patterns of results differ when we break down our sample by major. These figures show the results obtained using Model 2 for individuals in the top five most popular majors (based on last known enrollment) among students who were no longer enrolled.^9^ Figure 3 presents results for students who majored in the most popular major, liberal arts. Each subsequent figure shows the results for the next most popular major (health professions, business, law enforcement, and engineering technology, respectively). In all, these five majors cover 68.5% of the analytic sample. The results suggest that there is some variation in the returns to the different types of credits (and in the interaction of credits and degree attainment) across majors. However, it is sometimes difficult to draw strong conclusions, because of imprecision. The estimates for bachelor’s degree recipients are particularly imprecise because very few individuals obtained a bachelor’s degree within the follow-up period (only 5%).

For liberal arts majors, who comprise over a third of the sample, the returns on wages look fairly similar to patterns in the full sample—in terms of direction of relationships—but with a few notable exceptions. Although the relationships between academic credits and earnings appear largely positive, only non-completers appear to receive a significant boost from those credits (the line has a much tighter confidence interval than for the other degree attainment statuses and the slope of the line is the steepest which means the per-credit return is the greatest). Additionally, while certificate earners in the full sample appeared to experience negative returns to academic credits, the relationship appears null among liberal arts certificate earners. Developmental and technical credits appear positively related to both earnings and employment among liberal arts majors, mirroring the full sample. The relationship between technical credits at technical colleges and earnings seem strongest among certificate earners. Academic credits negatively predict employment for all credential earners (like in the full sample), but non-completers experience a small positive relationship between academic credits and employment—for each additional credit, they see a 0.10-percentage-point increase in the probability of employment (Fig. 3 and Online Appendix Table C1: β = 0.001, *SE* = 0.000, *p* < 0.001).

The patterns of returns for health professional majors (see Fig. 4) largely follow the same patterns as the full sample for developmental and technical credits, but illustrate a null relationship between academic credits and earnings (though negative, the confidence intervals largely overlap with zero). There is some variation across degree attainment groups in the earnings boost they receive from developmental math credits, where associate and bachelor’s completers appear to receive a larger boost than non-completers. Like-wise, non-completers seem to benefit less from technical credits from non-technical colleges than sub-baccalaureate credential holders. The patterns for the employment outcomes more closely align with the full sample, with a negative relationship between academic credits and employment and positive relationship between developmental and technical credits and employment. Other than the few patterns noted above for the earnings outcome, the returns to credits across different degree attainment groups appear more clustered together than what we observed for the full sample or among liberal arts majors. There may be greater overlap in experienced returns across degree attainment statuses—including non-completers—among health professional majors than in the full sample.

Figure 5 presents the results from Model 2 for a subsample of business majors, who comprise about 12 percent of the full sample. Business majors show a similar pattern of results, in terms of direction, for the relationship between the various credit types and both outcomes to those in the full sample, across different degree statuses. It is notable that the confidence interval overlaps with zero for the earning returns to developmental credits and the baseline starts below zero for most degree attainment statuses, which suggests that business students do not receive a boost from developmental credits, with the exception of non-completers (who do appear to experience a small positive relationship between credits and earnings for both developmental math and English).

Figure 6 illustrates that, among law enforcement majors, students appear to experience positive gains in wages for academic credits, developmental math and English credits, and technical credits at community colleges. Additionally, they appear to experience negative earnings returns to technical credits at technical colleges (perhaps because those credits do not correspond to their degree plan), where non-completers and associate degree recipients experience a 2.5% decrease in earnings for each additional technical credit earned at a technical college and certificate recipients experience a 1% decrease (see Table C4 in Online Appendix). This is distinct from the patterns for technical credits observed in all other presented majors. In general, the relationships between credits and employment also look flatter among law enforcement majors compared with the full sample. There is also a slight positive relationship between academic credits and employment for non-completers and certificate recipients, which differs from the full sample and other majors we highlight. Perhaps the flatter slopes reflect the fact that law enforcement students enter careers in which job training and work experience may be more important than academic training.

Finally, Fig. 7 presents the results for engineering technology majors, the fifth largest major in the analytic sample (full results available in Online Appendix Table C5). Engineering technology is primarily a field offering technical credentials. For that reason, bachelor’s degree receipt is particularly low within this major, so the confidence intervals are very large. Certificate recipients experience a negative relationship between academic credits and earnings, with a 0.8% decrease in earnings associated with each additional academic credit, though our estimates are noisy at the upper end of the credit distribution (likely because certificate earners in a technical program earn fewer academic credits). Other degree attainment statuses see a small positive relationship between academic credits and earnings. Technical credits appear to predict a boost in earnings for all degree attainment statuses groups, whether earned at a technical college or not. Similar to what we observed among health professional majors, the employment returns to developmental and technical credits are quite clustered together across the various degree attainment statuses. Academic credits appear to negatively predict employment among certificate and associate recipients, with null results for non-completers and bachelor’s degree recipients. Both types of developmental and technical credits appear to have a small positive relationship with employment and those patterns hold across the various degree attainment statuses.

Overall, the exercise of breaking out results by major aligns with prior research demonstrating variation in returns to education across field of study and moves beyond returns to a specific degree. Looking across the patterns of results from our major sub-analyses, we can highlight differential returns for non-completers across the different majors. Whereas the full sample of non-completers experience a negative relationship between academic credits and employment, some of the subsamples (liberal arts and law enforcement) actually experience a positive one. These differences suggest that even without a credential, students may find varied returns to the same types of credits across majors, where academic credits appear to be more valuable in some career paths than in others.

## Discussion

Most research focused on the returns to college among two-year college entrants focuses on the returns to credentials. We are aware of two studies that focus on the returns to credits: those of Bahr (2019), who examined variation in the returns to cumulative community college credits across majors, and of Hodara and Xu (2016), who examined the returns to developmental credits, in addition to those on academic and technical credits. In this study, we use administrative data from Texas and individual fixed effects models to examine variation in the returns to different types of college credits, including academic credits, technical credits, and developmental credits, and how those relationships vary across degree attainment status. We used two separate models. First, we relied on a model that controls for degree attainment, aligned with previous analyses in other states. Then, we used a model with interaction terms to explore variation in returns to credits by highest degree earned, and, from there, additional variation in patterns across last known major.

In the results from our first model, we see that, on average, Texas students who did not complete a credential experienced small negative returns (for both outcomes) to additional college-level academic credits and positive returns to additional developmental math and developmental English credits. The observed returns to developmental English credits align with Hodara and Xu’s (2016) results from community colleges in North Carolina and Virginia. They illustrated that the positive relationship between developmental education credits and earnings was primarily driven by the increase in probability of employment, which also appears to be the case in our data. Our findings about the returns to additional academic credits and developmental math credits contradict theirs: they found small positive returns to academic credits and negative returns to developmental math credits. Although we cannot know for certain why our results differ, it seems possible that this outcome is due to the different state contexts and timing. The data they used was from 2003 (in North Carolina) and 2006 (in Virginia) college entrants, which is well before some of the more recent “math pathways” reforms, in which colleges work to better align developmental and college-level math courses to students’ programs of study and career goals. It is possible that we capture the influence of math pathways reforms to developmental math that occurred during our cohort’s educational trajectory, given our timeframe of 2011–2018. Many colleges participated in collaborative efforts for math pathways—run by the Dana Center and Carnegie—by 2013 and 2014, though some incorporated homegrown efforts for math pathways prior to that (see Schudde & Keisler, 2019). It would be useful to see additional studies replicating these analyses to better understand the returns to different types of credits and how they may vary over time and context.

Our other major takeaway from Model 1 is that there are large returns to earning a degree, above and beyond the returns resulting from credits earned. This is particularly true for bachelor’s degree recipients. Although sub-baccalaureate credential recipients experience a boost from their credential, it seems possible that the negative returns to academic credits may at least partially capture negative returns for over-accumulating academic credits—when taking academic credit accumulation into account, the boost from an associate degree may diminish. Associate degree earners, for example, earned 79 academic credits on average (yet an associate degree requires only 60 credits).

Our subsequent analyses allowed us to further investigate heterogeneity within the returns to academic credits, which are the most prevalent credit type accrued, representing almost three-quarters of total credits accumulated by students in our analytic sample. Figure 1 offered greater insights into the negative relationship between academic credits and earnings among certificate recipients. Certificate experienced negative returns to academic credits (probably driving the average negative relationship between academic credits and employment outcomes), whereas non-completers, associate degree holders, and bachelor’s degree holders experienced small positive boost in earnings. Under the constraints of our first model, this variation was obscured by examining the average return to academic credits for all students.

Why would certificate earners experience negative effects of academic credits? As noted above, it seems likely that these negative relationships stem from taking unnecessary academic credits. Many students who earned those credentials may have been striving to earn a “higher” credential, meaning they accumulated academic credits beyond what was necessary for their degree. At the same time, it is also possible that many of those academic credits may not apply toward students’ degree plan, given high rates of major switching and misalignment between credits taken and degree requirements (Fink et al., 2018; Liu et al., 2020; Schudde, Ryu & Brown, 2020a, 2020b). Fink et al. (2018) show that introductory coursework, in particular, contribute to excess credits and argue that colleges should offer more structured pathways to avoid students taking additional introductory courses that will not contribute toward their desired degree. It is also possible that academic credits are not a strong signal of skills, where employers look more at the credential than at additional schooling through academic credits (which may not directly translate to marketable skills). That could partially explain the consistent negative association between academic credits and employment across all degree attainment statuses. Finally, it seems feasible that taking excess academic credits required students to forgo work experience that would otherwise have boosted their earnings.

The returns to additional technical credits varied substantially across credential attainment, although all the slopes were positive. We expected that technical credits might offer high returns without a degree in hand, because they may be more likely to contribute to skill development and therefore render a degree unnecessary. However, it appears that individuals with an associate degree receive the largest per-credit boost from technical credits. In terms of the slope (the per-credit change), certificate recipients received a smaller per-credit improvement even though their baseline (starting point with few credits) was higher. Individuals who earn a bachelor’s degree do not appear to benefit as much from technical credits.

The positive relationships between technical credits and both employment outcomes occurred whether credits were earned at technical colleges or not. As noted in “Methods” section, we identified technical credits using the WECM (standard course numbers for technical courses in Texas—which are delineated from academic courses in the ACGM). We distinguished between technical credits taken at technical colleges and community colleges because we anticipated differences in returns across the two institution types (the technical colleges in the state are funded based on labor market returns, incentivizing them to offer programs with stronger employment outcomes). There seemed to be somewhat more variation in the returns to technical credits for majors that were primarily offered as academic programs (e.g., liberal arts and law enforcement), with more consistent returns for majors more likely to results in a technical credential, such as engineering technology or health professional majors. At the same time, non-completers appeared to accrue very few technical credits from technical colleges, which may indicate higher completion rates at those institutions. It is possible that technical programs are more structured, particularly at institutions that specifically focus on technical credentials (which are primarily certificates and associate degrees). Because only technical credits with specific equivalents in the ACGM can transfer to academic programs, there may also be a natural set of guideposts that keep students in technical majors on track (they cannot easily switch to an academic major without losing the bulk of their credits) (THECB, 2020).

Prior literature explored the returns to credits (Bahr, 2019; Hodara & Xu, 2016), but extant studies did not allow for variation in credit returns across different levels of degree attainment, implicitly assuming parallel trends in returns across individual with different degree attainment. When we used a more flexible model that allowed variation in the returns to credits across highest credential earned, we were able to plot the heterogeneous returns to different types of credits across degree status. Overall, although our results provide confirming evidence that college credits provide positive returns for non-completers (e.g., Kane & Rouse, 1999; Grubb, 2002), our findings also illustrate that understanding the returns to credits among public two-year college students requires nuance—the returns depend on the type of credit *and* on the credential earned.

Our findings also provide some evidence to support prior evidence suggesting that non-completers in different majors and fields experience differential returns to credits (Bahr, 2019). Within our most popular majors, non-completers in health professions and business majors saw the smallest returns to academic credits while non-completers in engineering technology and liberal arts saw the largest returns. This result may speak to the fact that certain career fields are more likely to require a credential or a college degree than others. In the absence of a credential, accumulating academic credits may benefit only individuals in specific career paths.

At the same time, we recognize that students who accrued a large number of credits—especially academic credits—and are no longer enrolled may intend to return to college. One pressing need in the literature will be to continue following these cohorts of college entrants over time. Students, particularly those attending broad-access colleges, may take longer to complete their studies than our data allows us to capture. Attewell and Lavin (2007) found a substantial boost in degree attainment when they followed CUNY students for 10 years instead of 6 years (our current data allowed us to follow students for 7 years).

### Implications for Research and Practice

Across the country, public higher education systems—and community colleges in particular—are working to implement “guided pathways” reforms and to improve articulation policies (Bailey et al., 2015; Jenkins et al., 2020; Spencer, 2019). Guided pathways is a whole-college redesign model through which colleges backward map programs to baccalaureate transfer and good jobs while improving advising, instruction, and technology systems, all to enable students to select, plan, and complete programs more efficiently and affordably (Bailey et al., 2015; Jenkins et al., 2020). As colleges attempt wholescale restructuring, they need information about how students accrue credits and the implications of students’ credit-accrual patterns. Our findings, from the descriptive breakdown of across degree attainment statuses to the estimates of variation in returns to credits, offer insights to inform those efforts.

A common theme across our analyses was that academic credits do not positively predict earnings or employment (we generally found negative or null relationships between each additional academic credit and both employment outcomes). Academic coursework is often foundational in college degree programs—in that way they are difficult to disentangle from credentials themselves (which offer positive returns for credential completers). What our results illustrate is why it is prudent for students to avoid accruing more academic credits than are necessary for their degree. In Texas, all undergraduates in a degree program at public institutions must complete the state’s core curriculum, a set of general education coursework distributed across 8 component areas, including social and behavioral science, math, natural science, and humanities. Prior work in Texas illustrated that core credits earned at community colleges positively predict bachelor’s degree attainment among community college transfer students (Schudde et al., 2020a), but evidence from other state contexts suggests an association between introductory coursework (similar to the core) and excess credits. Taken together, the evidence from these studies and our results illustrates why colleges should focus efforts on avoiding excess academic credits.

Some states, including Texas, have implemented excess credit surcharges to discourage excess credits, yet research suggests that excess credit policies do not improve student outcomes and ultimately appear to increase student debt (Kramer et al., 2018). It may be more useful for colleges to instead focus their efforts on ensuring students determine their degree plan early on and select coursework accordingly. Additional advising and support structures to help students take credits strategically—with their preferred major and degree type in mind—could help boost student employment outcomes. These efforts could go hand-in-hand with ongoing guided pathways reforms.

Our results can help colleges consider the implications of whether and how different types of credits move across programs and the potential payoff of those credits in the labor market. For example, technical credits appear to improve earnings, but less so for some majors or degree types. Guidance for students should help them consider the implications of accumulating technical credits in an academic program and vice versa.

Replication and further exploration of this research can further illuminate variation in credit returns across programs and contexts to inform ongoing efforts to streamline college pathways. We hope to see additional studies illustrating the returns to different types of credits in various states to help make better sense of how these returns vary across contexts. In those efforts, researchers should take on the task of examining not only variation across types of credits, but also differential effects across degree attainment. Prior literature illustrates that the returns to credits and sub-baccalaureate credentials may vary widely across majors (Bahr, 2019; Dadgar & Trimble, 2014; Trimble & Xu, 2016; Turner, 2016), so additional exploration may be necessary in that area, especially in understanding differences across technical and academic majors.

## Supplementary Material

Supp Material 1

Supp Material 3

Supp Material 2

## Figures and Tables

**Table 1:** Variable names and descriptions

Variable name	Description

Dependent variables	
Quarterly earnings	Reported quarterly earnings from Texas Workforce Commission data for a given term, aligned with the 3 academic terms per year: spring, summer, fall (which includes Q3 and Q4 quarterly earnings combined)
Employment	Dichotomous measure of employment status in each term, aligned with the 3 academic terms per year, where the value is 1 in semesters in which the UI data reports non-zero earnings and 0 otherwise
Independent variables	
Cumulative academic credits	Cumulative number of academic credits earned before the given term
Cumulative Dev-Ed Math Credits	Cumulative number of developmental mathematics credits earned before the given term
Cumulative Dev-Ed English Credits	Cumulative number of developmental English credits earned before the given term
Cumulative Technical at Tech College	Cumulative number of credits earned before the given term in technical classes at a technical college
Cumulative Technical at Comm College	Cumulative number of credits earned before the given term in technical classes at a community college
Certificate	Indicator for whether or not the individual has earned a certificate prior to the given term
Associate degree	Indicator for whether or not the individual has earned an associate degree prior to the given term
Bachelor’s degree	Indicator for whether or not the individual has earned a bachelor’s degree prior to the given term
Highest degree earned	Indicator for the highest degree earned prior to the given term - no degree, certificate, associate degree, or bachelor’s degree (used in Model 2)
Time since award	A continuous variable that increases with the number of terms that have passed since the individual earned each award
Enrolled	Dummy variable capturing whether student is enrolled in given term, (= 1 if currently enrolled)
Enrolled credits	Total number of credits enrolled in during given term
Academic term	
Fall	Indicator for the given term being the fall semester
Spring	Indicator for the given term being the spring semester
Summer (reference)	Indicator for the given term being the summer semester
Ashenfelter Dip	
One Semester Prior to Enrollment	Indicator for term one semester prior to first enrollment
Two Semesters Prior to Enrollment	Indicator for term two semesters prior to first enrollment
Three Semesters Prior to Enrollment	Indicator for term three semesters prior to first enrollment
Year	Indicator for the given year

**Table 2:** Descriptive statistics for analytic samples

Variables	All public two-year entrants		Bachelor’s degree recipients		Associate degree recipients		Certificate recipients		Non-completers	
	Mean	SD	Mean	SD	Mean	SD	Mean	SD	Mean	SD

Race										
White	0.33	(0.47)	0.46	(0.50)	0.36	(0.48)	0.40	(0.49)	0.32	(0.47)
Black	0.19	(0.39)	0.08	(0.27)	0.12	(0.32)	0.12	(0.33)	0.21	(0.41)
Asian	0.02	(0.15)	0.04	(0.21)	0.03	(0.17)	0.01	(0.12)	0.02	(0.14)
Pacific Islander	<.01	(0.04)	<.01	(0.02)	<.01	(0.02)	<.01	(0.02)	<.01	(0.04)
Native American	<.01	(0.06)	<.01	(0.04)	<.01	(0.05)	<.01	(0.07)	<.01	(0.06)
Hispanic	0.42	(0.49)	0.36	(0.48)	0.46	(0.50)	0.44	(0.50)	0.42	(0.49)
Two or more races	0.02	(0.15)	0.04	(0.20)	0.03	(0.17)	0.01	(0.12)	0.02	(0.15)
Unknown race	0.02	(0.13)	<.01	(0.04)	0.01	(0.09)	0.02	(0.12)	0.02	(0.13)
International	<.01	(0.07)	0.01	(0.08)	0.01	(0.09)	0.01	(0.08)	<.01	(0.07)
Female	0.53	(0.50)	0.57	(0.49	0.58	(0.49)	0.40	(0.49)	0.53	(0.50)
Age at entrance	22.65	(7.55)	20.24	(5.58)	22.47	(7.62)	25.14	(9.20)	22.70	(7.51)
Types of credits taken										
Any developmental math	0.51	(0.50)	0.39	(0.49)	0.61	(0.49)	0.32	(0.47)	0.52	(0.50)
Any developmental English	0.48	(0.50)	0.26	(0.44)	0.46	(0.50)	0.33	(0.47)	0.50	(0.50)
Any technical credits	0.38	(0.49)	0.21	(0.41)	0.60	(0.49)	0.98	(0.14)	0.33	(0.47)
Credits completed										
Academic	32.29	(40.83)	127.01	(28.76)	78.52	(39.92)	13.32	(19.90)	21.34	(28.13)
Developmental math	2.93	(3.71)	2.13	(3.23)	4.04	(4.20)	1.83	(3.26)	2.92	(3.67)
Developmental English	2.91	(4.86)	1.06	(2.72)	2.72	(5.10)	1.96	(4.21)	3.11	(4.95)
Technical at tech college	0.50	(4.80)	0.04	(1.04)	2.06	(11.08)	2.03	(9.11)	0.26	(3.01)
Technical at Comm College	6.09	(13.19)	2.06	(7.06)	15.92	(21.42)	35.10	(17.64)	3.64	(8.80)
Total	44.71	(42.62)	132.30	(29.21)	103.26	(30.44)	54.24	(26.65)	31.26	(30.46)
Attended technical college	0.02	(0.14)	<0.01	(0.07)	0.04	(0.19)	0.06	(0.23)	0.02	(0.13)
Total semesters enrolled	5.22	(4.37)	12.06	(2.83)	11.23	(3.39)	5.67	(3.28)	4.02	(3.51)
Credits per semester enrolled	8.56	(3.95)	10.97	(4.02)	9.19	(3.80)	9.57	(4.52)	7.78	(3.67)
Transferred to a 4 year	0.15	(0.35)	1.00	(0.00)	0.43	(0.49)	0.03	(0.16)	0.06	(0.24)
Semesters since last enrollment^a^	10.82	(6.80)	4.39	(2.62)	4.39	(4.35)	10.49	(5.83)	12.04	(6.61)
Credentials earned										
Certificate	0.06	(0.24)	0.01	(0.12)	0.16	(0.37)	1.00	(0.00)	N/A	N/A
Associate degree	0.12	(0.32)	0.42	(0.49)	1.00	(0.00)	N/A	N/A	N/A	N/A
Bachelor’s degree	0.05	(0.23)	1.00	(0.00)	N/A	N/A	N/A	N/A	N/A	N/A
N	91,759		5110		8698		4001		73,950	

The table presents means and standard deviations (in parentheses) for the full analytic sample and subgroups of the population based on highest degree earned. Cells with averages representing proportions below 0.01 are masked due to requirements by Texas law

a “Semesters since last enrolled” refers to the length of time—as of the final wave of follow-up data (summer 2018)—since individuals enrolled in college

**Table 3:** Average returns to different types of credits: regression results from Model 1

Variables	Ln(wage)	Employment

Academic credits	− 0.003*** (0.000)	− 0.003*** (0.000)
Developmental math credits	0.014*** (0.001)	0.013*** (0.000)
Developmental English credits	0.013*** (0.001)	0.012*** (0.000)
Technical at technical college credits	0.007*** (0.001)	0.003*** (0.000)
Technical at non-technical college credits	0.004*** (0.000)	0.002*** (0.000)
Certificate	0.128*** (0.011)	0.051*** (0.006)
Associate	0.022* (0.009)	− 0.000 (0.005)
Bachelor’s	0.380*** (0.015)	0.125*** (0.008)
Certificate × time since certificate	− 0.014*** (0.002)	− 0.007*** (0.001)
Associate × time since associate	− 0.013*** (0.002)	− 0.012*** (0.001)
Bachelor’s × time since bachelor’s	− 0.003 (0.004)	− 0.022*** (0.002)
Enrolled	0.089*** (0.003)	0.123*** (0.002)
Enrolled credits	− 0.023*** (0.000)	− 0.007*** (0.000)
Fall semester	0.030*** (0.001)	0.032*** (0.000)
Spring semester	− 0.039*** (0.001)	− 0.026*** (0.000)
One semester prior to enrollment	− 0.029*** (0.003)	0.175*** (0.002)
Two semesters prior to enrollment	− 0.091*** (0.003)	0.135*** (0.002)
Three semesters prior to enrollment	− 0.064*** (0.004)	0.073*** (0.001)
Constant	0.001*** (0.000)	0.000 (0.000)
N(students)	91,759	91,759
N(students-semesters)	1,799,636	2,661,011

The table presents coefficients, with standard errors in parentheses. The dependent variable is the natural log of quarterly wages in column (1) and employment in column (2). Each observation in this model is at the individual-semester level (includes every term in which an individual had earnings for the wage outcome and every term for the employment outcome). The model includes individual fixed effects and individual time trends and county of employment fixed effects. Standard errors are clustered at the individual level. Model parameters are described in “Analytic Strategy” section under Model 1***

* p < 0.05,

** p < 0.01,

*** p < 0.001

## Footnotes

1 Some community college districts in Texas are comprised of distinct campuses with their own leadership and FICE codes, which report data separately to the THECB; we use those FICE codes to identify where students are enrolled.

2 Most individuals in the analytic sample have at least one term that is missing wage data out of the potential 29 semesters worth of earnings. 91.7% of all individuals in the full sample are missing at least one semester of wages. It varies a little by degree attainment, but not much, with 91.5% of non-completers, 90.0% certificate, 92.0% associate degree, and 95.8% of bachelor’s degree recipients missing at least one term of wages. On average, individuals have 19.6 semesters of positive wages. That number is 19.6 for non-completers, 20.2 for certificate, 19.9 for associate, and 18.9 for bachelor’s degree recipients. These patterns align with the literature that suggests that bachelor’s degree recipients are somewhat more likely to not earn wages while enrolled and to migrate for subsequent employment (see Scott-Clayton and Wen (2019) for additional information). We further discuss the limitations associated with using UI data in the limitations section below.

3 There were some outliers in earnings values reported in the UI data, including one very high value ($3 million within one term) and a handful of people earning below $1 within a term. We created an updated logged wages measure in which we capped the upper and lower values to align with the first and 99^th^ percentile. The uncapped logged wage measure (mean = 8.15, SD = 1.13; 1^st^ percentile = 4.38, 99^th^ percentile = 10.04) and the capped measure were quite similar, where the only difference is that the standard deviation of the capped measure was lower (SD = 1.09). We tested if the results were sensitive to the inclusion of outliers, running analyses with the capped and uncapped logged wage outcome – they were not. Ultimately, we decided that the capped logged-wage measure, which addressed outliers, had more logical values and used that in our reported findings.

4 We used the same model for both outcomes. For the dichotomous outcome capturing whether the individual is employed, this serves as a linear probability model, which is in line with the prior literature.

5 We tested some specifications in which we instead used terms since last enrolled (rather than terms since award). However, the measure was highly correlated with the time-varying measure of enrollment status; when running the specification, the time since enrollment measure was omitted due to multicollinearity. Ultimately, we settled on using time since award (this approach is similar to that used by Xu and Trimble (2016)).

6 Returns to academic credits for non-completers is captured by the coefficient on academic credits (β = 0.001, *SE* = 0.000, *p* < 0.001; note that these are rounded). Returns to academic credits for those who earn an associate degree is the coefficient on academic credits + the coefficient on academic credits interacted with associate degree (β = − 0.000, *SE* = 0.000, *p* > 0.05). Returns to academic credits for those who earn a bachelor’s degree is the coefficient on academic credits + the coefficient on academic credits interacted with bachelor’s degree (β = 0.001, SE = 0.001, *p* > 0.05).

7 Returns to academic credits for those who earn a certificate is the coefficient on academic credits + the coefficient on academic credits interacted with certificate (β = − 0.004, *SE* = 0.001, *p* < 0.001).

8 Returns to academic credits for non-completers is captured by the coefficient on academic credits (β = − 0.001, *SE* = 0.000, *p* < 0.001; note that these are rounded). Returns to academic credits for those who earn a certificate is the coefficient on academic credits + the coefficient on academic credits interacted with certificate (β = − 0.003, *SE* = 0.000, *p* < 0.001). Returns to academic credits for those who earn an associate degree is the coefficient on academic credits + the coefficient on academic credits interacted with associate degree (β = − 0.001, *SE* = 0.000, *p* < 0.01). Returns to academic credits for those who earn a bachelor’s degree is the coefficient on academic credits + the coefficient on academic credits interacted with bachelor’s degree (β = − 0.001, SE = 0.001, *p* < 0.05).

9 Online Appendix B (Tables B3–B7) show results for students who are currently enrolled.
