# Supplementary material for: Heterogeneity in the Returns to Credits for Public Two-Year College Entrants
Source: Res High Educ. Author manuscript; Available in PMC 2024 Jul 12. (PMC11244730; doi:10.1007/s11162-021-09654-8)
Supplement: Supp Material 1 [file NIHMS1908692-supplement-Supp_Material_1.doc]

Appendix A

*Table A1
Average Returns to Different Types of Credits by Highest Degree: Results from Model 2*

|  | Ln(wage) |  | Employment |  |
| --- | --- | --- | --- | --- |
| Academic Credits | 0.001^***^ | (0.000) | -0.001^***^ | (0.000) |
| Developmental Math Credits | 0.017^***^ | (0.001) | 0.019^***^ | (0.000) |
| Developmental English Credits | 0.017^***^ | (0.000) | 0.016^***^ | (0.000) |
| Technical at Technical College Credits | 0.010^***^ | (0.001) | 0.005^***^ | (0.000) |
| Technical at non-Technical College Credits | 0.007^***^ | (0.000) | 0.005^***^ | (0.000) |
| Academic Credits x Enrolled | -0.003^***^ | (0.000) | -0.002^***^ | (0.000) |
| Academic Credits x Certificate | -0.004^***^ | (0.001) | -0.003^***^ | (0.000) |
| Academic Credits x Associate | -0.000 | (0.000) | -0.001^**^ | (0.000) |
| Academic Credits x Bachelors | -0.001 | (0.001) | -0.001^*^ | (0.001) |
| Academic Credits x Enrolled x Certificate | 0.003^***^ | (0.001) | 0.002^***^ | (0.000) |
| Academic Credits x Enrolled x Associate | 0.000 | (0.000) | 0.002^***^ | (0.000) |
| Academic Credits x Enrolled x Bachelors | -0.000 | (0.003) | 0.000 | (0.001) |
| Developmental Math Credits x Enrolled | -0.004^***^ | (0.001) | -0.011^***^ | (0.000) |
| Developmental Math Credits x Certificate | 0.002 | (0.004) | 0.001 | (0.003) |
| Developmental Math Credits x Associate | 0.010^***^ | (0.002) | 0.008^***^ | (0.001) |
| Developmental Math Credits x Bachelors | 0.001 | (0.003) | 0.007^***^ | (0.002) |
| Developmental Math Credits x Enrolled x Certificate | 0.004 | (0.005) | 0.007^***^ | (0.003) |
| Developmental Math Credits x Enrolled x Associate | -0.000 | (0.003) | 0.005^***^ | (0.001) |
| Developmental Math Credits x Enrolled x Bachelors | -0.018 | (0.021) | -0.011 | (0.010) |
| Developmental English Credits x Enrolled | -0.005^***^ | (0.000) | -0.006^***^ | (0.000) |
| Developmental English Credits x Certificate | -0.002 | (0.003) | -0.006^***^ | (0.002) |
| Developmental English Credits x Associate | 0.002 | (0.002) | -0.003^***^ | (0.001) |
| Developmental English Credits x Bachelors | -0.006 | (0.005) | -0.001 | (0.003) |
| Developmental English Credits x Enrolled x Certificate | 0.008 | (0.004) | 0.009^***^ | (0.002) |
| Developmental English Credits x Enrolled x Associate | 0.006^**^ | (0.002) | 0.006^***^ | (0.001) |
| Developmental English Credits x Enrolled x Bachelors | -0.032 | (0.023) | 0.005 | (0.009) |
| Technical at Tech College x Enrolled | -0.010^***^ | (0.001) | -0.006^***^ | (0.000) |
| Technical at Tech College x Certificate | -0.001 | (0.001) | -0.002^**^ | (0.001) |
| Technical at Tech College x Associate | 0.004^***^ | (0.001) | 0.001 | (0.001) |
| Technical at Tech College x Bachelors | -0.012 | (0.013) | -0.001 | (0.007) |
| Technical at Tech College x Enrolled x Certificate | 0.005^*^ | (0.002) | 0.005^***^ | (0.001) |
| Technical at Tech College x Enrolled x Associate | 0.003 | (0.003) | 0.005^***^ | (0.001) |
| Technical at Tech College x Enrolled x Bachelors | 0.010 | (0.014) | -0.003 | (0.007) |
| Technical at non-Tech College x Enrolled | -0.005^***^ | (0.000) | -0.005^***^ | (0.000) |
| Technical at non-Tech College x Certificate | -0.000 | (0.001) | -0.003^***^ | (0.000) |
| Technical at non-Tech College x Associate | 0.006^***^ | (0.001) | 0.001^***^ | (0.000) |
| Technical at non-Tech College x Bachelors | -0.002 | (0.002) | -0.001 | (0.001) |
| Technical at non-Tech College x Enrolled x Certificate | 0.004^***^ | (0.001) | 0.005^***^ | (0.001) |
| Technical at non-Tech College x Enrolled x Associate | 0.003^***^ | (0.001) | 0.004^***^ | (0.000) |
| Technical at non-Tech College x Enrolled x Bachelors | 0.001 | (0.006) | -0.003 | (0.002) |
| Highest Degree = Certificate | 0.170^***^ | (0.032) | 0.155^***^ | (0.019) |
| Highest Degree = Associate | -0.115^***^ | (0.031) | -0.065^***^ | (0.018) |
| Highest Degree = Bachelors | 0.133 | (0.125) | 0.117^*^ | (0.067) |
| Certificate x Time since Certificate | -0.008^***^ | (0.002) | -0.003^***^ | (0.001) |
| Associate x Time since Associate | -0.012^***^ | (0.002) | -0.014^***^ | (0.001) |
| Bachelors x Time since Bachelors | 0.013^**^ | (0.004) | 0.014^***^ | (0.002) |
| Enrolled | 0.015^***^ | (0.003) | 0.165^***^ | (0.002) |
| Certificate x Enrolled | -0.144^**^ | (0.045) | -0.214^***^ | (0.023) |
| Associate x Enrolled | 0.046 | (0.038) | -0.114^***^ | (0.019) |
| Bachelors x Enrolled | 0.253 | (0.426) | 0.139 | (0.202) |
| Fall Semester | 0.020^***^ | (0.001) | -0.034^***^ | (0.000) |
| Spring Semester | -0.047^***^ | (0.001) | 0.026^***^ | (0.000) |
| One Semester Prior to Enrollment | 0.010^**^ | (0.003) | 0.204^***^ | (0.002) |
| Two Semesters Prior to Enrollment | -0.054^***^ | (0.003) | 0.160^***^ | (0.002) |
| Three Semesters Prior to Enrollment | -0.027^***^ | (0.004) | 0.098^***^ | (0.002) |
| Constant | -0.000^***^ | (0.000) | 0.000^***^ | (0.000) |
| N(students) | 91,759 |  | 91,759 |  |
| N(student-semesters) | 1,799,636 |  | 2,661,011 |  |

*Notes:* The table presents coefficients, with standard errors in parentheses. The dependent variable is the natural log of quarterly wages in column (1) and employment in column (3). Each observation in this model is at the individual-semester level (includes every term in which an individual had earnings for the wage outcome and every term for the employment outcome). The model includes individual fixed effects, individual time trends, and county of employment fixed effects. Standard errors are clustered at the individual level. The model parameters are described in the Analytic Strategy section under Model 2.

^*^p<0.05, ^**^p<0.01, ^***^p<0.001
