# Supplementary material for: Heterogeneity in the Returns to Credits for Public Two-Year College Entrants
Source: Res High Educ. Author manuscript; Available in PMC 2024 Jul 12. (PMC11244730; doi:10.1007/s11162-021-09654-8)
Supplement: Supp Material 3 [file NIHMS1908692-supplement-Supp_Material_3.doc]

Appendix C

Full Regression Results for Model 2 by Major

*Table C1
Average Returns to Different Types of Credits by Highest Degree: Liberal Arts Majors*

| Liberal Arts Majors | Ln(wage) |  | Employment |  |
| --- | --- | --- | --- | --- |
| Academic Credits | 0.003^***^ | (0.000) | 0.001^***^ | (0.000) |
| Developmental Math Credits | 0.018^***^ | (0.001) | 0.020^***^ | (0.001) |
| Developmental English Credits | 0.015^***^ | (0.001) | 0.015^***^ | (0.001) |
| Technical at Technical College Credits | 0.004 | (0.005) | 0.007^**^ | (0.003) |
| Technical at non-Technical College Credits | 0.008^***^ | (0.001) | 0.005^***^ | (0.001) |
| Academic Credits x Enrolled | -0.002^***^ | (0.000) | -0.002^***^ | (0.000) |
| Academic Credits x Certificate | -0.002 | (0.001) | -0.001 | (0.001) |
| Academic Credits x Associate | -0.001 | (0.001) | -0.002^**^ | (0.001) |
| Academic Credits x Bachelors | -0.004 | (0.003) | -0.004^*^ | (0.002) |
| Academic Credits x Enrolled x Certificate | 0.002 | (0.002) | 0.002 | (0.001) |
| Academic Credits x Enrolled x Associate | -0.001 | (0.001) | 0.003^***^ | (0.001) |
| Academic Credits x Enrolled x Bachelors | 0.009 | (0.005) | 0.004 | (0.003) |
| Developmental Math Credits x Enrolled | -0.006^***^ | (0.001) | -0.012^***^ | (0.001) |
| Developmental Math Credits x Certificate | -0.003 | (0.010) | 0.011 | (0.007) |
| Developmental Math Credits x Associate | 0.015^***^ | (0.005) | 0.008^**^ | (0.003) |
| Developmental Math Credits x Bachelors | -0.012 | (0.018) | -0.001 | (0.007) |
| Developmental Math Credits x Enrolled x Certificate | 0.015 | (0.010) | 0.017^***^ | (0.005) |
| Developmental Math Credits x Enrolled x Associate | -0.008 | (0.006) | 0.010^**^ | (0.003) |
| Developmental Math Credits x Enrolled x Bachelors | 0.041 | (0.036) | -0.012 | (0.014) |
| Developmental English Credits x Enrolled | -0.005^***^ | (0.001) | -0.006^***^ | (0.000) |
| Developmental English Credits x Certificate | 0.004 | (0.010) | -0.003 | (0.006) |
| Developmental English Credits x Associate | -0.003 | (0.005) | -0.003 | (0.003) |
| Developmental English Credits x Bachelors | -0.012 | (0.022) | -0.007 | (0.012) |
| Developmental English Credits x Enrolled x Certificate | -0.011 | (0.013) | 0.013^**^ | (0.005) |
| Developmental English Credits x Enrolled x Associate | -0.005 | (0.006) | 0.004 | (0.003) |
| Developmental English Credits x Enrolled x Bachelors | -0.108 | (0.066) | 0.034 | (0.025) |
| Technical at Tech College x Enrolled | -0.001 | (0.003) | -0.004^*^ | (0.002) |
| Technical at Tech College x Certificate | 0.016^**^ | (0.005) | -0.002 | (0.004) |
| Technical at Tech College x Associate | 0.010 | (0.006) | 0.002 | (0.003) |
| Technical at Tech College x Enrolled x Certificate | 0.007 | (0.005) | 0.011 | (0.006) |
| Technical at Tech College x Enrolled x Associate | -0.010 | (0.006) | -0.004 | (0.004) |
| Technical at non-Tech College x Enrolled | -0.006^***^ | (0.001) | -0.006^***^ | (0.000) |
| Technical at non-Tech College x Certificate | -0.000 | (0.002) | -0.001 | (0.001) |
| Technical at non-Tech College x Associate | 0.005^***^ | (0.001) | 0.002 | (0.001) |
| Technical at non-Tech College x Bachelors | -0.021^*^ | (0.009) | -0.005 | (0.005) |
| Technical at non-Tech College x Enrolled x Certificate | 0.007^**^ | (0.003) | 0.005^***^ | (0.001) |
| Technical at non-Tech College x Enrolled x Associate | 0.004^*^ | (0.002) | 0.005^***^ | (0.001) |
| Technical at non-Tech College x Enrolled x Bachelors | 0.022^*^ | (0.010) | 0.005 | (0.005) |
| Highest Degree = Certificate | 0.131 | (0.073) | 0.120^*^ | (0.052) |
| Highest Degree = Associate | -0.071 | (0.075) | -0.021 | (0.047) |
| Highest Degree = Bachelors | 0.713 | (0.414) | 0.529^*^ | (0.239) |
| Certificate x Time since Certificate | -0.016^*^ | (0.006) | -0.004 | (0.004) |
| Associate x Time since Associate | 0.000 | (0.005) | -0.017^***^ | (0.003) |
| Bachelors x Time since Bachelors | 0.001 | (0.020) | -0.025^*^ | (0.012) |
| Enrolled | 0.028^***^ | (0.005) | 0.191^***^ | (0.003) |
| Certificate x Enrolled | -0.232^*^ | (0.118) | -0.242^***^ | (0.055) |
| Associate x Enrolled | 0.117 | (0.110) | -0.178^**^ | (0.056) |
| Bachelors x Enrolled | -1.098 | (0.745) | -0.372 | (0.388) |
| Fall Semester | 0.020^***^ | (0.002) | -0.033^***^ | (0.001) |
| Spring Semester | -0.049^***^ | (0.002) | 0.023^***^ | (0.001) |
| One Semester Prior to Enrollment | 0.014^*^ | (0.006) | 0.220^***^ | (0.003) |
| Two Semesters Prior to Enrollment | -0.051^***^ | (0.006) | 0.170^***^ | (0.003) |
| Three Semesters Prior to Enrollment | -0.025^***^ | (0.006) | 0.108^***^ | (0.003) |
| Constant | 0.001^***^ | (0.000) | 0.000^***^ | (0.000) |
| N(students) | 31,859 |  | 31,859 |  |
| N(student-semesters) | 625,910 |  | 923,911 |  |

*Notes:* The table presents coefficients, with standard errors in parentheses. The results were obtained by running Model 2 on a subgroup of students who were identified as having a liberal arts major in the enrollment data. The dependent variable is the natural log of quarterly wages in column (1) and employment in column (2). Each observation in this model is at the individual-semester level (includes every term in which an individual had earnings for the wage outcome and every term for the employment outcome). The model includes individual fixed effects and individual time trends and county of employment fixed effects. Standard errors are clustered at the individual level. The model parameters are described in the Analytic Strategy section under Model 2.

*p<0.05, **p<0.01, ***p<0.001

*Table C2
Average Returns to Different Types of Credits by Highest Degree: Health Professions Majors*

| Health Professions Majors | Ln(wage) |  | Employment |  |
| --- | --- | --- | --- | --- |
| Academic Credits | -0.000 | (0.000) | -0.001^***^ | (0.000) |
| Developmental Math Credits | 0.009^***^ | (0.002) | 0.014^***^ | (0.001) |
| Developmental English Credits | 0.018^***^ | (0.002) | 0.018^***^ | (0.001) |
| Technical at Technical College Credits | 0.006 | (0.003) | 0.004^*^ | (0.002) |
| Technical at non-Technical College Credits | 0.007^***^ | (0.001) | 0.004^***^ | (0.000) |
| Academic Credits x Enrolled | -0.003^***^ | (0.000) | -0.002^***^ | (0.000) |
| Academic Credits x Certificate | -0.005^***^ | (0.001) | -0.003^***^ | (0.001) |
| Academic Credits x Associate | -0.002 | (0.001) | -0.003^***^ | (0.001) |
| Academic Credits x Bachelors | 0.000 | (0.004) | -0.002 | (0.002) |
| Academic Credits x Enrolled x Certificate | 0.002 | (0.002) | 0.002^*^ | (0.001) |
| Academic Credits x Enrolled x Associate | -0.001 | (0.001) | 0.002^**^ | (0.001) |
| Academic Credits x Enrolled x Bachelors | -0.014^**^ | (0.005) | -0.001 | (0.003) |
| Developmental Math Credits x Enrolled | -0.004^*^ | (0.002) | -0.011^***^ | (0.001) |
| Developmental Math Credits x Certificate | 0.009 | (0.010) | 0.013 | (0.007) |
| Developmental Math Credits x Associate | -0.001 | (0.007) | 0.014^***^ | (0.004) |
| Developmental Math Credits x Bachelors | 0.022 | (0.023) | 0.004 | (0.009) |
| Developmental Math Credits x Enrolled x Certificate | 0.002 | (0.013) | -0.004 | (0.006) |
| Developmental Math Credits x Enrolled x Associate | 0.005 | (0.009) | 0.006 | (0.004) |
| Developmental Math Credits x Enrolled x Bachelors | -0.034 | (0.036) | 0.005 | (0.022) |
| Developmental English Credits x Enrolled | -0.006^***^ | (0.001) | -0.006^***^ | (0.001) |
| Developmental English Credits x Certificate | -0.005 | (0.009) | 0.002 | (0.007) |
| Developmental English Credits x Associate | -0.006 | (0.007) | -0.003 | (0.003) |
| Developmental English Credits x Bachelors | -0.014 | (0.024) | 0.016 | (0.010) |
| Developmental English Credits x Enrolled x Certificate | -0.000 | (0.010) | -0.001 | (0.005) |
| Developmental English Credits x Enrolled x Associate | 0.008 | (0.007) | 0.010^**^ | (0.003) |
| Developmental English Credits x Enrolled x Bachelors | -0.084 | (0.051) | 0.028 | (0.039) |
| Technical at Tech College x Enrolled | -0.015^***^ | (0.003) | -0.009^***^ | (0.002) |
| Technical at Tech College x Certificate | -0.005 | (0.004) | -0.002 | (0.003) |
| Technical at Tech College x Associate | 0.002 | (0.004) | 0.002 | (0.003) |
| Technical at Tech College x Bachelors | -0.018 | (0.010) | -0.010^*^ | (0.004) |
| Technical at Tech College x Enrolled x Certificate | -0.005 | (0.007) | 0.005 | (0.004) |
| Technical at Tech College x Enrolled x Associate | 0.017^***^ | (0.006) | 0.007^*^ | (0.003) |
| Technical at Tech College x Enrolled x Bachelors | 0.011 | (0.016) | 0.011 | (0.007) |
| Technical at non-Tech College x Enrolled | -0.013^***^ | (0.001) | -0.006^***^ | (0.000) |
| Technical at non-Tech College x Certificate | 0.002 | (0.002) | 0.000 | (0.001) |
| Technical at non-Tech College x Associate | 0.008^***^ | (0.001) | 0.001 | (0.001) |
| Technical at non-Tech College x Bachelors | -0.000 | (0.005) | -0.004 | (0.002) |
| Technical at non-Tech College x Enrolled x Certificate | 0.007^**^ | (0.002) | 0.003^**^ | (0.001) |
| Technical at non-Tech College x Enrolled x Associate | 0.011^**^ | (0.002) | 0.005^***^ | (0.001) |
| Technical at non-Tech College x Enrolled x Bachelors | -0.007 | (0.011) | -0.001 | (0.005) |
| Highest Degree = Certificate | 0.240^**^ | (0.081) | 0.099^*^ | (0.050) |
| Highest Degree = Associate | 0.100 | (0.103) | 0.097 | (0.064) |
| Highest Degree = Bachelors | 0.165 | (0.582) | 0.102 | (0.231) |
| Certificate x Time since Certificate | -0.015^**^ | (0.005) | -0.003 | (0.003) |
| Associate x Time since Associate | 0.012^*^ | (0.006) | -0.001 | (0.003) |
| Bachelors x Time since Bachelors | 0.006 | (0.018) | -0.027^**^ | (0.010) |
| Enrolled | -0.000 | (0.008) | 0.133^***^ | (0.005) |
| Certificate x Enrolled | 0.047 | (0.105) | -0.064 | (0.046) |
| Associate x Enrolled | 0.050 | (0.132) | -0.208^**^ | (0.069) |
| Bachelors x Enrolled | 1.936^**^ | (0.673) | 0.197 | (0.468) |
| Fall Semester | 0.023^***^ | (0.003) | -0.025^***^ | (0.001) |
| Spring Semester | -0.046^***^ | (0.003) | 0.031^***^ | (0.001) |
| One Semester Prior to Enrollment | 0.003 | (0.009) | 0.172^***^ | (0.005) |
| Two Semesters Prior to Enrollment | -0.014 | (0.009) | 0.145^***^ | (0.004) |
| Three Semesters Prior to Enrollment | -0.004 | (0.009) | 0.103^***^ | (0.004) |
| Constant | 0.002^***^ | (0.000) | 0.000^***^ | (0.000) |
| N(students) | 12,148 |  | 12,148 |  |
| N(student-semesters) | 244,409 |  | 352,292 |  |

*Notes:* The table presents coefficients, with standard errors in parentheses. The results were obtained by running Model 2 on a subgroup of students who were identified as having a health professions major in the enrollment data. The dependent variable is the natural log of quarterly wages in column (1) and employment in column (2). Each observation in this model is at the individual-semester level (includes every term in which an individual had earnings for the wage outcome and every term for the employment outcome).The model includes individual fixed effects and individual time trends and county of employment fixed effects. Standard errors are clustered at the individual level. The model parameters are described in the Analytic Strategy section under Model 2.

*p<0.05, **p<0.01, ***p<0.001

*Table C3
Average Returns to Different Types of Credits by Highest Degree: Business Majors*

| Business Majors | Ln(wage) |  | Employment |  |
| --- | --- | --- | --- | --- |
| Academic Credits | 0.001 | (0.000) | -0.002^***^ | (0.000) |
| Developmental Math Credits | 0.020^***^ | (0.002) | 0.018^***^ | (0.001) |
| Developmental English Credits | 0.018^***^ | (0.002) | 0.012^***^ | (0.001) |
| Technical at Technical College Credits | 0.010^*^ | (0.005) | 0.003 | (0.003) |
| Technical at non-Technical College Credits | 0.005^***^ | (0.001) | 0.005^***^ | (0.001) |
| Academic Credits x Enrolled | -0.003^***^ | (0.000) | -0.002^***^ | (0.000) |
| Academic Credits x Certificate | -0.003 | (0.002) | -0.002 | (0.001) |
| Academic Credits x Associate | 0.000 | (0.001) | -0.001 | (0.000) |
| Academic Credits x Bachelors | -0.003 | (0.002) | -0.001 | (0.001) |
| Academic Credits x Enrolled x Certificate | 0.003 | (0.003) | 0.002 | (0.001) |
| Academic Credits x Enrolled x Associate | 0.000 | (0.001) | 0.002^***^ | (0.000) |
| Academic Credits x Enrolled x Bachelors | -0.004 | (0.014) | -0.005 | (0.003) |
| Developmental Math Credits x Enrolled | -0.004^**^ | (0.002) | -0.010^***^ | (0.001) |
| Developmental Math Credits x Certificate | 0.002 | (0.014) | 0.001 | (0.007) |
| Developmental Math Credits x Associate | 0.011^*^ | (0.005) | 0.007^*^ | (0.003) |
| Developmental Math Credits x Bachelors | -0.008 | (0.008) | 0.008 | (0.005) |
| Developmental Math Credits x Enrolled x Certificate | 0.012 | (0.016) | 0.008 | (0.007) |
| Developmental Math Credits x Enrolled x Associate | 0.009 | (0.005) | 0.007^*^ | (0.003) |
| Developmental Math Credits x Enrolled x Bachelors | 0.008 | (0.068) | 0.004 | (0.030) |
| Developmental English Credits x Enrolled | -0.004^***^ | (0.001) | -0.005^***^ | (0.001) |
| Developmental English Credits x Certificate | 0.011 | (0.012) | -0.006 | (0.005) |
| Developmental English Credits x Associate | 0.001 | (0.003) | -0.003 | (0.002) |
| Developmental English Credits x Bachelors | -0.009 | (0.008) | -0.002 | (0.005) |
| Developmental English Credits x Enrolled x Certificate | -0.001 | (0.010) | 0.009^*^ | (0.004) |
| Developmental English Credits x Enrolled x Associate | 0.005 | (0.003) | 0.004 | (0.002) |
| Developmental English Credits x Enrolled x Bachelors | -0.038 | (0.143) | 0.098 | (0.083) |
| Technical at Tech College x Enrolled | -0.008^***^ | (0.004) | -0.001 | (0.002) |
| Technical at Tech College x Certificate | 0.009 | (0.008) | -0.000 | (0.004) |
| Technical at Tech College x Associate | 0.004 | (0.006) | 0.009^***^ | (0.002) |
| Technical at Tech College x Enrolled x Certificate | 0.006 | (0.006) | -0.001 | (0.003) |
| Technical at Tech College x Enrolled x Associate | -0.011 | (0.011) | -0.003 | (0.003) |
| Technical at non-Tech College x Enrolled | -0.001^*^ | (0.001) | -0.004^***^ | (0.000) |
| Technical at non-Tech College x Certificate | 0.003 | (0.003) | -0.002 | (0.002) |
| Technical at non-Tech College x Associate | 0.004^**^ | (0.001) | 0.000 | (0.001) |
| Technical at non-Tech College x Bachelors | -0.002 | (0.004) | 0.001 | (0.002) |
| Technical at non-Tech College x Enrolled x Certificate | 0.001 | (0.003) | 0.005^**^ | (0.002) |
| Technical at non-Tech College x Enrolled x Associate | -0.002 | (0.002) | 0.003^**^ | (0.001) |
| Technical at non-Tech College x Enrolled x Bachelors | -0.001 | (0.014) | -0.007 | (0.004) |
| Highest Degree = Certificate | -0.018^**^ | (0.122) | 0.070 | (0.059) |
| Highest Degree = Associate | -0.157^*^ | (0.072) | -0.034 | (0.040) |
| Highest Degree = Bachelors | 0.485 | (0.285) | 0.120 | (0.231) |
| Certificate x Time since Certificate | 0.010 | (0.006) | 0.013^**^ | (0.004) |
| Associate x Time since Associate | -0.019^***^ | (0.004) | -0.015^***^ | (0.003) |
| Bachelors x Time since Bachelors | 0.014 | (0.008) | -0.016^***^ | (0.004) |
| Enrolled | 0.030^***^ | (0.008) | 0.149^***^ | (0.004) |
| Certificate x Enrolled | -0.096 | (0.143) | -0.228^***^ | (0.066) |
| Associate x Enrolled | 0.102 | (0.074) | -0.139^***^ | (0.040) |
| Bachelors x Enrolled | 0.642 | (1.674) | 0.751 | (0.431) |
| Fall Semester | 0.014^***^ | (0.004) | -0.035^***^ | (0.001) |
| Spring Semester | -0.051^***^ | (0.003) | 0.022^***^ | (0.001) |
| One Semester Prior to Enrollment | 0.018 | (0.010) | 0.179^***^ | (0.005) |
| Two Semesters Prior to Enrollment | -0.066^***^ | (0.009) | 0.144^***^ | (0.005) |
| Three Semesters Prior to Enrollment | -0.038^***^ | (0.010) | 0.094^***^ | (0.004) |
| Constant | -0.000^*^ | (0.000) | 0.000^***^ | (0.000) |
| N(students) | 11,056 |  | 11,056 |  |
| N(student-semesters) | 222,383 |  | 320,624 |  |

*Notes:* The table presents coefficients, with standard errors in parentheses. The results were obtained by running Model 2 on a subgroup of students who were identified as having a business major. The dependent variable is the natural log of quarterly wages in column (1) and employment in column (2). Each observation in this model is at the individual-semester level (includes every term in which an individual had earnings for the wage outcome and every term for the employment outcome). The model includes individual fixed effects and individual time trends and county of employment fixed effects. Standard errors are clustered at the individual level. The model parameters are described in the Analytic Strategy section under Model 2.

*p<0.05, **p<0.01, ***p<0.001

*Table C4
Average Returns to Different Types of Credits by Highest Degree: Law Enforcement Majors*

| Law Enforcement Majors | Ln(wage) |  | Employment |  |
| --- | --- | --- | --- | --- |
| Academic Credits | 0.002^***^ | (0.001) | 0.001^***^ | (0.000) |
| Developmental Math Credits | 0.010^**^ | (0.004) | 0.019^***^ | (0.002) |
| Developmental English Credits | 0.024^***^ | (0.003) | 0.023^***^ | (0.001) |
| Technical at Technical College Credits | -0.025^**^ | (0.008) | -0.003 | (0.003) |
| Technical at non-Technical College Credits | 0.009^***^ | (0.002) | 0.006^***^ | (0.001) |
| Academic Credits x Enrolled | -0.002^***^ | (0.000) | -0.002^***^ | (0.000) |
| Academic Credits x Certificate | -0.005^*^ | (0.002) | -0.001 | (0.001) |
| Academic Credits x Associate | -0.001 | (0.001) | 0.000 | (0.001) |
| Academic Credits x Bachelors | 0.005 | (0.004) | 0.002 | (0.003) |
| Academic Credits x Enrolled x Certificate | 0.004^*^ | (0.002) | 0.002 | (0.001) |
| Academic Credits x Enrolled x Associate | -0.000 | (0.002) | 0.001 | (0.001) |
| Academic Credits x Enrolled x Bachelors | -0.071 | (0.057) | -0.005 | (0.005) |
| Developmental Math Credits x Enrolled | -0.005 | (0.003) | -0.011^***^ | (0.001) |
| Developmental Math Credits x Certificate | -0.016 | (0.016) | -0.015^*^ | (0.007) |
| Developmental Math Credits x Associate | 0.014 | (0.008) | 0.001 | (0.006) |
| Developmental Math Credits x Bachelors | 0.222 | (0.016) | 0.016 | (0.009) |
| Developmental Math Credits x Enrolled x Certificate | -0.006 | (0.013) | 0.011 | (0.007) |
| Developmental Math Credits x Enrolled x Associate | -0.004 | (0.010) | 0.006 | (0.005) |
| Developmental Math Credits x Enrolled x Bachelors | 0.253^***^ | (0.074) | 0.218^***^ | (0.022) |
| Developmental English Credits x Enrolled | -0.007^***^ | (0.002) | -0.011^***^ | (0.001) |
| Developmental English Credits x Certificate | -0.008 | (0.013) | -0.006 | (0.007) |
| Developmental English Credits x Associate | -0.000 | (0.007) | -0.002 | (0.004) |
| Developmental English Credits x Bachelors | -0.013 | (0.017) | -0.006 | (0.008) |
| Developmental English Credits x Enrolled x Certificate | 0.011 | (0.010) | 0.017^**^ | (0.005) |
| Developmental English Credits x Enrolled x Associate | 0.011 | (0.008) | 0.010^*^ | (0.004) |
| Technical at Tech College x Enrolled | 0.003 | (0.003) | -0.002 | (0.001) |
| Technical at Tech College x Certificate | 0.014 | (0.008) | 0.010 | (0.008) |
| Technical at Tech College x Associate | 0.000 | (0.008) | 0.008^**^ | (0.003) |
| Technical at Tech College x Enrolled x Certificate | -0.009 | (0.005) | -0.001 | (0.002) |
| Technical at Tech College x Enrolled x Associate | 0.002 | (0.006) | 0.001 | (0.002) |
| Technical at non-Tech College x Enrolled | -0.010^***^ | (0.001) | -0.006^***^ | (0.001) |
| Technical at non-Tech College x Certificate | -0.007^*^ | (0.003) | -0.007^***^ | (0.001) |
| Technical at non-Tech College x Associate | -0.001 | (0.003) | -0.001 | (0.002) |
| Technical at non-Tech College x Bachelors | -0.003 | (0.008) | -0.000 | (0.003) |
| Technical at non-Tech College x Enrolled x Certificate | 0.012^***^ | (0.003) | 0.007^***^ | (0.001) |
| Technical at non-Tech College x Enrolled x Associate | 0.010^**^ | (0.004) | 0.006^***^ | (0.002) |
| Technical at non-Tech College x Enrolled x Bachelors | -0.030 | (0.033) | 0.002 | (0.004) |
| Highest Degree = Certificate | 0.355^***^ | (0.100) | 0.225^***^ | (0.047) |
| Highest Degree = Associate | -0.026 | (0.115) | -0.076 | (0.069) |
| Highest Degree = Bachelors | -0.704 | (0.565) | -0.299 | (0.336) |
| Certificate x Time since Certificate | 0.005 | (0.007) | -0.003 | (0.004) |
| Associate x Time since Associate | -0.011 | (0.008) | -0.011^**^ | (0.004) |
| Bachelors x Time since Bachelors | 0.044^*^ | (0.018) | -0.001 | (0.008) |
| Enrolled | -0.008 | (0.012) | 0.201^***^ | (0.007) |
| Certificate x Enrolled | -0.171 | (0.118) | -0.183^***^ | (0.054) |
| Associate x Enrolled | 0.004 | (0.162) | -0.134^*^ | (0.066) |
| Bachelors x Enrolled | 9.061 | (6.973) | 0.742 | (0.625) |
| Fall Semester | 0.025^***^ | (0.005) | -0.039^***^ | (0.002) |
| Spring Semester | -0.043^***^ | (0.005) | 0.022^***^ | (0.002) |
| One Semester Prior to Enrollment | 0.007 | (0.015) | 0.235^***^ | (0.007) |
| Two Semesters Prior to Enrollment | -0.061^***^ | (0.015) | 0.192^***^ | (0.007) |
| Three Semesters Prior to Enrollment | -0.042^***^ | (0.016) | 0.101^***^ | (0.006) |
| Constant | -0.000 | (0.000) | 0.000^***^ | (0.000) |
| N(students) | 5,087 |  | 5,087 |  |
| N(student-semesters) | 102,022 |  | 147,523 |  |

*Notes:* The table presents coefficients, with standard errors in parentheses. The results were obtained by running Model 2 on a subgroup of students who were identified as having a law enforcement major. The dependent variable is the natural log of quarterly wages in column (1) and employment in column (2). Each observation in this model is at the individual-semester level (includes every term in which an individual had earnings for the wage outcome and every term for the employment outcome). The model includes individual fixed effects and individual time trends and county of employment fixed effects. Standard errors are clustered at the individual level. The model parameters are described in the Analytic Strategy section under Model 2. *p<0.05, **p<0.01, ***p<0.001

*Table C5
Average Returns to Different Types of Credits by Highest Degree: Engineering Technology Majors*

| Engineering Technology Majors | Ln(wage) |  | Employment |  |
| --- | --- | --- | --- | --- |
| Academic Credits | 0.006^***^ | (0.001) | 0.002 | (0.001) |
| Developmental Math Credits | 0.020^***^ | (0.006) | 0.015^***^ | (0.003) |
| Developmental English Credits | 0.016^***^ | (0.005) | 0.017^***^ | (0.002) |
| Technical at Technical College Credits | 0.010^***^ | (0.002) | 0.003^**^ | (0.001) |
| Technical at non-Technical College Credits | 0.004^***^ | (0.001) | 0.003^***^ | (0.001) |
| Academic Credits x Enrolled | -0.004^***^ | (0.001) | -0.002^***^ | (0.000) |
| Academic Credits x Certificate | -0.014^*^ | (0.006) | -0.003 | (0.002) |
| Academic Credits x Associate | -0.003 | (0.002) | -0.002^*^ | (0.001) |
| Academic Credits x Bachelors | -0.000 | (0.009) | 0.006 | (0.004) |
| Academic Credits x Enrolled x Certificate | 0.008 | (0.006) | -0.000 | (0.003) |
| Academic Credits x Enrolled x Associate | 0.005 | (0.003) | 0.004^**^ | (0.001) |
| Developmental Math Credits x Enrolled | -0.008^*^ | (0.004) | -0.007^**^ | (0.002) |
| Developmental Math Credits x Certificate | -0.004 | (0.014) | -0.009 | (0.008) |
| Developmental Math Credits x Associate | -0.007 | (0.015) | 0.012 | (0.006) |
| Developmental Math Credits x Bachelors | 0.014 | (0.050) | -0.017 | (0.031) |
| Developmental Math Credits x Enrolled x Certificate | 0.013 | (0.015) | 0.018 | (0.010) |
| Developmental Math Credits x Enrolled x Associate | 0.016 | (0.026) | -0.020 | (0.011) |
| Developmental English Credits x Enrolled | -0.001 | (0.003) | -0.006^***^ | (0.002) |
| Developmental English Credits x Certificate | -0.012 | (0.007) | -0.008^*^ | (0.003) |
| Developmental English Credits x Associate | 0.008 | (0.014) | -0.000 | (0.007) |
| Developmental English Credits x Bachelors | -0.006 | (0.025) | 0.008 | (0.011) |
| Developmental English Credits x Enrolled x Certificate | 0.013 | (0.013) | 0.011 | (0.008) |
| Developmental English Credits x Enrolled x Associate | -0.040^*^ | (0.018) | -0.008 | (0.006) |
| Technical at Tech College x Enrolled | -0.011^***^ | (0.002) | -0.005^***^ | (0.001) |
| Technical at Tech College x Certificate | 0.013^**^ | (0.004) | 0.001 | (0.002) |
| Technical at Tech College x Associate | 0.002 | (0.003) | -0.000 | (0.002) |
| Technical at Tech College x Enrolled x Certificate | 0.004 | (0.005) | 0.007^*^ | (0.003) |
| Technical at Tech College x Enrolled x Associate | 0.009 | (0.010) | 0.007^**^ | (0.003) |
| Technical at non-Tech College x Enrolled | -0.002^*^ | (0.001) | -0.002^***^ | (0.000) |
| Technical at non-Tech College x Certificate | 0.011^***^ | (0.003) | -0.001 | (0.002) |
| Technical at non-Tech College x Associate | 0.002 | (0.004) | -0.001 | (0.002) |
| Technical at non-Tech College x Bachelors | 0.003 | (0.014) | -0.002 | (0.007) |
| Technical at non-Tech College x Enrolled x Certificate | -0.008^*^ | (0.004) | 0.000 | (0.002) |
| Technical at non-Tech College x Enrolled x Associate | 0.005 | (0.005) | 0.004^*^ | (0.002) |
| Highest Degree = Certificate | -0.235^*^ | (0.102) | 0.055 | (0.065) |
| Highest Degree = Associate | 0.143 | (0.208) | -0.001 | (0.102) |
| Highest Degree = Bachelors | 0.271 | (1.217) | -0.864 | (0.611) |
| Certificate x Time since Certificate | -0.021^***^ | (0.006) | -0.008^*^ | (0.004) |
| Associate x Time since Associate | -0.018^*^ | (0.008) | -0.013^**^ | (0.004) |
| Bachelors x Time since Bachelors | 0.005 | (0.029) | -0.017 | (0.014) |
| Enrolled | -0.029 | (0.017) | 0.103^***^ | (0.009) |
| Certificate x Enrolled | 0.198 | (0.157) | -0.009 | (0.082) |
| Associate x Enrolled | -0.249 | (0.294) | -0.186 | (0.144) |
| Fall Semester | 0.015^*^ | (0.007) | -0.031^***^ | (0.002) |
| Spring Semester | -0.048^***^ | (0.007) | 0.036^***^ | (0.002) |
| One Semester Prior to Enrollment | 0.027 | (0.020) | 0.180^***^ | (0.010) |
| Two Semesters Prior to Enrollment | -0.057^**^ | (0.015) | 0.147^***^ | (0.009) |
| Three Semesters Prior to Enrollment | -0.014 | (0.020) | 0.073^***^ | (0.009) |
| Constant | -0.002^**^ | (0.000) | 0.000^***^ | (0.000) |
| N(students) | 2,705 |  | 2,705 |  |
| N(student-semesters) | 54,376 |  | 78,445 |  |

*Notes:* The table presents coefficients, with standard errors in parentheses. The results were obtained by running Model 2 on a subgroup of students who were identified as having an engineering technology major in the enrollment data. The dependent variable is the natural log of quarterly wages in column (1) and employment in column (2). Each observation in this model is at the individual-semester level (includes every term in which an individual had earnings for the wage outcome and every term for the employment outcome). The model includes individual fixed effects and individual time trends and county of employment fixed effects. Standard errors are clustered at the individual level. The model parameters are described in the Analytic Strategy section under Model 2.

*p<0.05, **p<0.01, ***p<0.001
