# Supplementary material for: Heterogeneity in the Returns to Credits for Public Two-Year College Entrants
Source: Res High Educ. Author manuscript; Available in PMC 2024 Jul 12. (PMC11244730; doi:10.1007/s11162-021-09654-8)
Supplement: Supp Material 2 [file NIHMS1908692-supplement-Supp_Material_2.docx]

Appendix B

Results from Model 2 for Currently Enrolled Students

**Fig. B1** *Average Returns to Different Types of Credits by Highest Degree for Enrolled Students: Model 2 Results for Earnings.* This figure presents predicted earnings (in logged quarterly earnings) with 95% confidence intervals, obtained using the results from Model 2 for students currently enrolled. For full regression results, see Appendix A, Table A1 (this figure makes use of the interaction terms). N(students) = 91,759; N(student-semesters) = 1,799,636.

**Fig. B2** *Average Returns to Different Types of Credits by Highest Degree for Enrolled Students: Model 2 Results for Employment Status.* This figure presents predicted employment with 95% confidence intervals, obtained using the results from Model 2 for students currently enrolled. For full regression results, see Appendix A, Table A1 (this figure makes use of the interaction terms). N(students) = 91,759; N(student-semesters) = 2,661,011.

**Fig. B3** *Liberal Arts Majors: Average Returns to Different Types of Credits by Highest Degree for Enrolled Students.* This figure presents predicted earnings (in logged quarterly earnings) (left panel) and predicted employment (right panel) with 95% confidence intervals, obtained using the results from Model 2 for students currently enrolled. The analysis was run on a subgroup of students who were identified as having a liberal arts major. For full regression results, see Appendix C, Table C1 (this figure makes use of the interaction terms). For regression on earnings: N(students) = 31,859; N(student-semesters) = 625,910. For regression on employment: N(students) = 31,859; N(student-semesters) = 923,911.

**Fig. B4** *Health Professional Majors: Average Returns to Different Types of Credits by Highest Degree for Enrolled Students*. This figure presents predicted earnings (in logged quarterly earnings) (left panel) and predicted employment (right panel) with 95% confidence intervals, obtained using the results from Model 2 for students currently enrolled. The analysis was run on a subgroup of students who were identified as having a health professions major. For full regression results, see Appendix C, Table C2 (this figure makes use of the interaction terms). For regression on earnings: N(students) = 12,148; N(student-semesters) = 244,409. For regression on employment: N(students) = 12,148; N(student-semesters) = 352,292.

**Fig. B5** *Business Majors: Average Returns to Different Types of Credits by Highest Degree for Enrolled Students*. This figure presents predicted earnings (in logged quarterly earnings) (left panel) and predicted employment (right panel) with 95% confidence intervals, obtained using the results from Model 2 for students currently enrolled. The analysis was run on a subgroup of students who were identified as having a business major. For full regression results, see Appendix C, Table C3 (this figure makes use of the interaction terms). For regression on earnings: N(students) = 11,056; N(student-semesters) = 222,383. For regression on employment: N(students) = 11,056; N(student-semesters) = 320,624.

**Fig. B6** *Law Enforcement Majors: Average Returns to Different Types of Credits by Highest Degree for Enrolled Students*. This figure presents predicted earnings (in logged quarterly earnings) (left panel) and predicted employment (right panel) with 95% confidence intervals, obtained using the results from Model 2 for students currently enrolled. The analysis was run on a subgroup of students who were identified as having a law enforcement major. For full regression results, see Appendix C, Table C4 (this figure makes use of the interaction terms). Fore regression on earnings: N(students) = 5,087; N(student-semesters) = 102,022. For regression on employment: N(students) = 5,087; N(student-semesters) = 147,523.

**Fig. B7** *Engineering Technology Majors: Average Returns to Different Types of Credits by Highest Degree for Enrolled Students*. This figure presents predicted earnings (in logged quarterly earnings) (left panel) and predicted employment (right panel) with 95% confidence intervals, obtained using the results from Model 2 for students currently enrolled. The analysis was run on a subgroup of students who were identified as having an engineering technology major. For full regression results, see Appendix C, Table C5 (this figure makes use of the interaction terms). For regression on earnings: N(students) = 2,705; N(student-semesters) = 54,376. For regression on employment: N(students) = 2,705; N(student-semesters) = 78,445.
